# Steric and Electronic Effects in the Enzymatic Catalysis of Choline-TMA Lyase

**DOI:** 10.3390/biom16010037

**Published:** 2025-12-25

**Authors:** Valentin Gogonea, Stanley L. Hazen

**Affiliations:** 1 Department of Heart, Blood & Kidney Research, Cleveland Clinic, Cleveland, OH 44195, USA; hazens@ccf.org; 2 Department of Chemistry, Cleveland State University, Cleveland, OH 44115, USA; 3 Center for Microbiome and Human Health, Cleveland Clinic, Cleveland, OH 44195, USA; 4 Department of Cardiovascular Medicine, Heart, Vascular and Thoracic Institute, Cleveland Clinic, Cleveland, OH 44195, USA; 5 Department of Molecular Medicine, Cleveland Clinic Lerner College of Medicine, Case Western Reserve University, Cleveland, OH 44195, USA

**Keywords:** intestinal microbes, cardiometabolic disease, choline utilization cluster (CutC) enzyme, glycyl radical enzyme, choline, carbinolamine, trimethylamine, acetaldehyde, QM/MM calculations, reaction mechanism

## Abstract

An important microbial enzymatic pathway in the gut is choline/phosphatidylcholine degradation in which intestinal microbes utilize dietary choline to produce TMA via the choline utilization cluster polypeptide C (CutC) enzyme, a member of the glycyl radical enzyme family. Our goal in this theoretical work was to study the reaction mechanism and elucidate how the enzyme environment (steric and electronic) modulates the reaction path. Dissecting the effect of the enzyme environment on the reaction mechanism and shedding light on how steric and electronic effects affect the reaction path is an insightful and significant contribution of this work. Our theoretical results suggest that the final product of enzyme catalysis might be carbinolamine and not TMA and acetaldehyde. In addition, we found out that Glu_491_ plays the role of a base in this reaction (a disputed fact) by temporarily abstracting a proton from the hydroxylic group of choline sometime during the reaction—with the proton transfer being critical for the reaction to proceed to completion. We also found that the choice of computational protocol not only alters the reaction energetics but can change the reaction path by creating new intermediates and transition states or eliminating existing ones.

## 1. Introduction

There is a growing appreciation that the gut microbiome significantly contributes to human health and diseases, including cardiometabolic disorders [1]. Namely, bioactive metabolites (e.g., trimethylamine (TMA), short-chain fatty acids, aromatic amino acid metabolites, etc.) are synthesized by gut microbes from dietary nutrients (e.g., choline, carnitine, carbohydrates, proteins, etc.) which, following adsorption into the host, either in native form or following host metabolic modification, can act as circulating hormones, impacting physiological processes and disease susceptibility in the host [1]. Therefore, a holistic view of metabolism seems to emerge in which both the host and the intestinal microbiota contribute to the metabolic profile of an individual and play a significant role in human health. Collectively, the gut microbiota thus act as an endocrine organ that signals to distal organs through a myriad of small molecules which modulate complex physiological pathways in the host and ultimately contribute to phenotypes like inflammation, insulin resistance, obesity, and cardiovascular disease (CVD) [1].

**Scheme 1:**
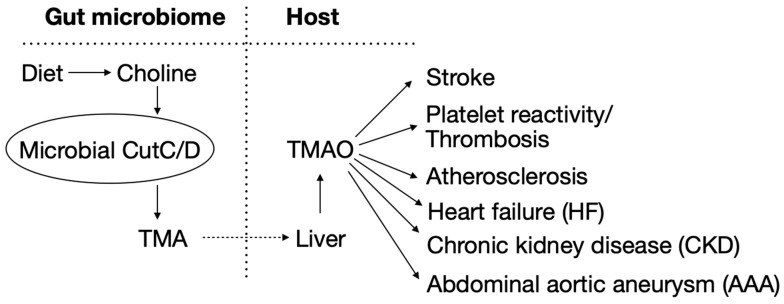
Graphic illustrating choline degradation to TMA by gut microbes, oxidation to TMAO in the host, and the involvement of TMAO in human health.

An important microbial enzymatic pathway in the gut is choline/phosphatidylcholine degradation in which intestinal microbes utilize dietary choline to produce TMA [2,3,4] via the choline utilization gene cluster (cut) polypeptide C (CutC), the catalytically active enzyme, and CutD, an activating polypeptide [5]. Following absorption into mammalian hosts via portal circulation, TMA is further metabolized in the liver by flavin-dependent monooxygenases (FMOs, especially FMO3) into trimethylamine N-oxide (TMAO) [2,6,7,8] (Scheme 1). A substantial body of evidence shows TMAO is both clinically associated with and mechanistically linked (through animal model studies) to atherosclerosis [2,3,9,10,11,12,13,14,15,16,17], thrombosis [8,18,19,20], heart failure [21,22,23,24,25], renal dysfunction [26,27,28], aortic aneurysm [29], stroke [30,31], and even obesity [32,33,34] (Scheme 1). Accordingly, the reduction in TMAO production by the inhibition of gut microbial enzyme CutC [10,19] through small molecule inhibitors is of considerable interest [1]. To design more effective CutC inhibitors, it is thus of interest to have improved detailed understanding of the reaction mechanism of microbial choline-TMA lyase.

Choline TMA lyases are anaerobic bacterial enzymes that convert choline into TMA and acetaldehyde [5,35,36]. Gut microbial CutC is considered a major contributor to TMA and, thus, TMAO production in both omnivore and vegans alike because of the abundance of phosphatidylcholine in bile [37]. However, omnivorous dietary patterns have been shown to further enhance TMAO generation from potential alternative pathways (via CntA/B, YeaW/X, and gbuA enzymes) utilizing alternative dietary nutrients including carnitine and its gut microbial product, γ-butyrobetaine [9,38,39,40]. In general, animal model studies have shown that the non-lethal inhibition of the bacterial enzyme CutC can serve as a promising approach to selectively target the contribution of the gut microbial TMAO pathway to cardiometabolic diseases in the host [10,19,23,27,41]. In particular, the non-lethal targeting of gut microbiota TMA lyase activity can effectively be used in the treatment of atherosclerotic heart disease [19]; therefore, enzymes producing TMA (e.g., CutC/D, CntA/B, YeaW/X, gbuA) have recently become targets for both scientific investigation and drug development [10,19,23,27,41,42].

CutC is a member of the glycyl radical enzyme family—a large and diverse group of enzymes capable of degrading pyruvate [43], ribonucleotide [44], glycerol [45], *trans*-4-hydroxy-L-proline [46], and isethionate [47] and synthesizing benzylsuccinate [48]. Two X-ray crystal structures of microbial CutCs have been reported thus far—one for *K. pneumoniae* CutC [35] and another for *D. alaskensis* CutC [36]. Earlier theoretical work aimed at resolving the reaction mechanism for choline-TMA lyase by the gut microbial enzyme CutC used the crystal structure of *D. alaskensis* CutC (PDB: 5FAU) [36] for theoretical investigations. Roberts et al. [19] reported the first calculations for the reaction mechanism of choline-TMA lyase by using a quantum mechanical (QM) system formed from choline, a methylthiol radical, and an acetate ion as substitutes for the Cys_489_ radical and Glu_491_ in *D. alaskensis* CutC (PDB: 5FAU). Combined quantum mechanical–molecular mechanics (QM/MM) calculations on CutC enzyme were recently reported by Yang et al. [49], Yang et al. [50], and Hanzevacki et al. [51].

Our goal in this theoretical study was to elucidate how the enzyme environment (steric and electronic effects) modulates the reaction mechanism of choline-TMA lyase in light of the unexpected findings by the recently published QM/MM calculations that concluded that TMA and acetaldehyde are not the final products of the enzyme activity [50,51]. This conclusion contradicts a rather entrenched understanding of the activity of this enzyme [5,35,36] and the similarity in catalysis with other enzymes [52,53,54]. To understand this unexpected result of theoretical calculations (in line with theoretical prediction for other enzymes utilizing radical chemistry [55]), we first mapped out the reaction mechanism of a QM system formed of choline and surrogate chemical compounds that mimic essential amino acid residues from the CutC active site and compared this reaction mechanism with those obtained by QM/MM either with or without the molecular mechanics (MM) partial atomic charges embedded in the QM system. Using this computational protocol, we were able to split the effect of the enzyme environment on the reaction into steric (cage) and electronic (polarization). We analyzed the impact of these effects on the reaction, and the results of these analyses are presented.

## 2. Methods

### 2.1. Quantum Mechanical Calculations

The quantum mechanical (QM) cluster calculations in vacuum were performed on a QM system composed of choline (substrate) surrounded by the chemical surrogates of three amino acid residues from the enzyme’s active site (PDB id: 5FAU [36]), i.e., Asp_216_, Cys_489_ radical, and Glu_491_. The three residues had their backbone amino and carbonyl groups replaced with H atoms, producing a QM system with 52 atoms (named CholineActiveSite-1); the C_α_ atoms of these chemical surrogates were constrained to their crystal structure positions (PDB id: 5FAU) during geometry optimization and transition state search. To start, we used the ab initio Hartree–Fock method [56] with Pople’s split-valence double-zeta basis set 6-31G(d) [57], as implemented in the program Gaussian16 [58], to make initial explorations (vacuum calculations) of the potential energy surface (PES) of choline-TMA lyase within the environment of the chemical surrogates (Asp_216_, Cys_489_ radical, and Glu_491_) mimicking essential reactive features of the CutC active site.

These lower-level calculations provided not only a preliminary PES and a reaction mechanism for this simplified chemical environment of choline but also starting geometries for intermediates and transition states to use in PES explorations utilizing more sophisticated QM methods. All species identified along the reaction pathway (reactant, product, intermediates, transition states) were verified to be stationary points on the PES by performing frequency calculations and confirming that intermediates have no imaginary frequency and transition states have one. We performed intrinsic reaction coordinate (IRC) calculations on each transition state (TS) to determine the intermediates that link to the TS, and ensure that the reaction path is continuous from reactant to product. We also performed ab initio Hartree–Fock/6-31G(d) calculations on larger QM systems of 141 (CholineActiveSite-2) and 202 atoms (CholineActiveSite-3).

To refine the PES mapped at the Hartree–Fock/6-31G(d) level of ab initio theory, we selected three density functional theory (DFT) methods: B3LYP [59,60], M06-2X [61], and ωB97XD [62] and their variants, including dispersion interactions corrections [63,64] (B3LYP-D3, M06-2X-D3; ωB97XD uses a version of Grimme’s D2 dispersion model [63,64]) and Ahlrichs’ split-valence double-zeta basis set Def2-SVP [65,66]. We used these three DFT functionals to perform vacuum cluster calculations on CholineActiveSite-1 only.

### 2.2. Combined Quantum Mechanical–Molecular Mechanics Calculations

The exploration of the PES for the choline-TMA lyase reaction in the enzyme environment by combined quantum mechanical–molecular mechanics (QM/MM) calculations was performed using only the ωB97XD functional [62] with Ahlrichs’ split-valence double-zeta basis set Def2-SVP [65,66]. A *D. alaskensis* CutC monomer with its crystallization water molecules were extracted from the PDB entry 5FAU [36] and used to build the QM/MM system as follows. Because the crystal structure of *D. alaskensis* CutC (5FAU) has hydrogen atoms assigned, we decided to use this assignment in our QM/MM calculations. While it is customary to assign protonation states for acid and base amino acids using an empirical method as implemented in PROPKA [67] and H++ [68] programs, we decided not to alter the protonation states of the amino acids from the ones assigned in the crystal structure based on the following argument: the protonation states of residues Asp_216_, Cys_489_, and Glu_491_ are most relevant from the point of view of the reaction mechanism as these three residues are directly involved in the reaction. Our investigation suggests that Glu_491_ is a weak base that accepts a proton from choline at some point during the reaction, and to do so, Glu_491_ cannot be protonated at the beginning of the reaction. Asp_216_ is in close vicinity to the (positively charged) trimethylammonium head of choline, and we concluded that there is a high chance that the negatively charged Asp_216_ is not protonated in order to engage in a strong electrostatic interaction with the positively charged choline’s trimethylammonium head. In addition, the proton from choline’s OH might end up on TMA via Asp_216_ to produce protonated TMA. This will not be possible if Asp_216_ is protonated at the beginning of the reaction. Cys_489_ is a radical at the beginning of the reaction, and we did not consider the abstraction of a H atom from Cys_489_ by the glycyl radical Gly_821_. The protonation states of the rest of the amino acids outside the enzyme active site probably have little effect on the reaction mechanism as these amino acids are not directly involved in the reaction.

First, to remove bad contacts between non-bonded atoms in the crystal structure, the enzyme system (CutC monomer with its crystallization water) was energy minimized using the Amber99SB force field [69] as implemented in the molecular dynamics simulation program Gromacs [70]. Next, CholineActiveSite-1 (choline, Asp_216_, Cys_489_ radical, Glu_491_) was used as the QM subsystem of the QM/MM system, where the backbone amino and carbonyl groups of these amino acids were replaced by H link atoms. The ONIOM method [71], as implemented in the program Gaussian16 [58], was used for all QM/MM calculations, in which the Amber force field [72] was utilized to describe the interactions of the enzyme atoms and crystallization water molecules in the molecular mechanics (MM) subsystem of the QM/MM system. Amino acid residues and water molecules beyond 3.5 Å of CholineActiveSite-1 were kept frozen during geometry optimization and transition state search.

Transition states (TS) and reaction intermediates successfully located by the TS search algorithm were verified to be stationary points on the PES by performing frequency calculations. We used intrinsic reaction coordinate (IRC) calculations to connect these transition states with nearby reaction intermediates (or reactant and product). Not all transition states could be located by the search algorithm. Unfortunately, many attempts to locate certain transition states ended in an unrecoverable micro-iteration error. In these cases, we performed a manual search along a (most likely) reaction coordinate (usually a distance between two atoms) by multistep constrained geometry optimizations.

## 3. Results

### 3.1. Selecting the Size of the QM System

In an earlier attempt to establish the reaction mechanism of choline lyase by CutC enzyme, we used the program Gamess [73] to perform quantum mechanical (QM) calculations in vacuum at the Hartree–Fock/6-31G(d) level of ab initio theory [56] on a molecular system composed of choline and active site residues from *D. alaskensis* CutC, Cys_489_ and Glu_491_ (PDB id: 5FAU [36]), in which we replaced Cys_489_ with CH_3_-S (methylthiol radical) and Glu_491_ with the acetate anion [19]. With this system we obtained a reaction pathway composed of three short-lived intermediates and four transition states [19], shown in Supplementary Figure S1. Also shown are the potential energy surface (PES), the activation energy for each transition state, and the reaction energies of the intermediates and the product (TMA + acetaldehyde + thiol radical + acetic acid) relative to the reactant (choline + thiol radical + acetate ion).

Our goals in the present study were two-fold: first, to upgrade the calculation methodology by using the density functional theory (DFT) and more sophisticated basis sets (e.g., triple-zeta basis sets augmented with polarization and diffusion basis functions); second, to analyze the steric (cage) and electronic (polarization) effect that the enzyme environment (CutC) has on the reaction mechanism by adopting a quantum mechanical–molecular mechanics (QM/MM) strategy in which the enzyme/crystal water environment are explicitly included into the calculation of the PES of the reaction. In this way, we can shed light on how the enzyme environment modulates the reaction mechanism of choline-TMA lyase and how the knowledge obtained from this analysis might be used in other applications regarding this enzymatic reaction—like the design of CutC inhibitors through structure activity relationships (SAR) approaches [19].

To use the QM/MM approach in this theoretical study, we had to first select a region of the enzyme active site to be described by the QM methodology. Keeping in mind that the QM system must contain, at a minimum, the ligand and any amino acid residue involved in the reaction directly by bond breaking/bond formation, we defined three putative QM regions in *D. alaskensis* CutC (PDB id: 5FAU [36], Supplementary Figure S2) which contain, in addition to choline, several amino acids (from CutC active site) closest to choline and named these QM regions the following: CholineActiveSite-1 (52 atoms) comprising choline and residues Asp_216_, Cys_489_, and Glu_491_; CholineActiveSite-2 (141 atoms) comprising CholineActiveSite-1 and Tyr_208_, Phe_389_, Phe_395_, Thr_502_, Tyr_506_, and a crystal water molecule (residue index in the PDB file = 1072); and CholineActiveSite-3 (202 atoms) comprising CholineActiveSite-2 and Thr_334_, Met_487_, Leu_698_, and Ile_700_. In each QM system, the “terminal” amide and carbonyl groups of the amino acid backbone were replaced by hydrogen atoms, making a C_α_ methyl group. To avoid unrealistic migrations of the residues from their position in the enzyme during geometry optimization or transition state search, the C_α_ atoms were frozen to their position in the crystal structure. Constraining the C_α_ atoms to the positions they have in the crystal structure when performing QM cluster calculations can be thought of as a steric constraint as it mimics, to some extent, the presence of the enzyme cavity that hosts the active-site amino acids that the ligand interacts with.

To see if the size of the QM system used in calculations affects the reaction mechanism in a significant way, i.e., by altering the chemical identity of the intermediates or transition states, or by shortening/lengthening the reaction pathway, we mapped the full reaction pathway for each QM system (CholineActiveSite-1,2,3) using cluster calculations in vacuum at the HF/6-31G(d) level of ab initio theory. The reaction mechanism and the associated kinetic/thermochemical data (activation energies, relative reaction energies) for each QM system are shown in Supplementary Figure S3. It is interesting to note that the reaction pathways shown in Supplementary Figures S1 and S3 are very similar, with the same intermediates and transition states, even if the QM system used in this study is much bigger. There are some differences in the kinetics/thermochemistry of the reaction, which is to be expected as the environment of choline changes from one QM system to another; however, the order of magnitude of the kinetics/thermochemistry data for individual transition states and intermediates is preserved among the QM systems of different sizes. This similarity in reaction pathways shows that the interaction of choline with the cysteinyl radical (Cys_489_) and glutamate (Glu_491_) defines both the essential features of the mechanism of choline lyase by CutC (i.e., the reaction steps) and the identity of the transition states and intermediates at this level of quantum mechanics (HF/6-31G(d)). The similarity of these reaction pathways suggests that the other residues in the active site only participate in the fine-tuning of the kinetics and thermochemistry of the reaction.

The QM cluster calculations at the HF level of ab initio theory (Supplementary Figure S3) suggest that the first step in the reaction is the transfer of a H atom from choline (bonded to C_1_) to the Cys_489_ (ethylthiol) radical, which seems to be the rate-limiting step with an activation energy of ~29 kcal/mol. In the next step, a proton is transferred from choline radical to the glutamate residue (butyrate ion), the C-N bond is cleaved in the third step, and the H atom is transferred back from cysteine to the acetaldehyde radical in the fourth step, which has the second largest activation energy (~25 kcal/mol) in the reaction pathway. To capture the proton from the glutamic acid residue in the fifth step, the resulting TMA needs to rotate and orient in such a way that the lone electron pair of the N atom points towards the OH group of the glutamic acid residue. The reaction is overall exothermic, with the product (protonated TMA + acetaldehyde + ethylthiol radical + butyrate ion) being ~25 kcal/mol more stable than the reactant (choline + ethylthiol radical + butyrate ion).

### 3.2. Selecting the DFT Functional for Exploring the Reaction Mechanism

**Figure 1:**
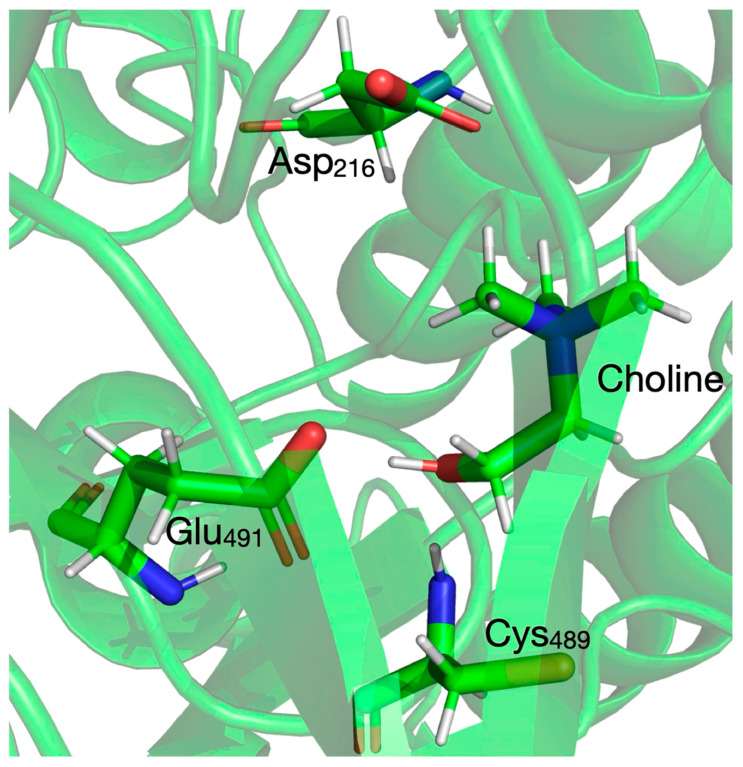
The active site of the *D. alaskensis* CutC enzyme (PDB id: 5FAU) showing residues (Asp_216_, Cys_489_, Glu_491_) involved in bond-breaking and bond formation with choline during the reaction of degrading choline into TMA and acetaldehyde. The three active site residues are drawn with sticks (carbon = green, oxygen = red, nitrogen = blue, hydrogen = white) while, for clarity, the backbone of the rest of the protein is shown as a faded ribbon. The crystallization water is not shown. For the QM cluster calculations in vacuum, the terminal amino and carbonyl groups of the residues were substituted with H atoms and the C_α_ atoms were frozen during geometry optimizations or transition state searches. This QM system (52 atoms) is formed of choline and chemical surrogates for Asp_216_, Cys_489_, and Glu_491_.

While keeping in mind that the QM/MM calculations are much more demanding in computational resources than the QM cluster calculations in vacuum performed on molecular configurations with the same size/composition of the QM system, we decided to use CholineActiveSite-1 as a QM system for all subsequent calculations. In the exploratory calculations presented above, we used the Hartree–Fock (HF) Hamiltonian because it is faster than the more sophisticated density functional theory (DFT) approach (which, compared to HF, includes electron correlation), and the configurations of the transition states and intermediates can be used as starting geometries for further potential energy surface (PES) explorations. For refining the PES, we selected one of the many DFT functionals [74,75,76,77,78] available in familiar quantum chemistry programs like Gaussian16 [58]. As a basis set, we decided to use Ahlrichs’ split-valence double-zeta basis set augmented with polarization functions (Def2-SVP [65,66]). The region of the *D. alaskensis* CutC crystal structure with choline bound and the three residues of the CholineActiveSite-1 region (displayed as sticks) are shown in Figure 1. The protein backbone is shown as a semi-transparent ribbon. For cluster vacuum calculations, CholineActiveSite-1 region contains chemical surrogates for Asp_216_, Cys_489_ radical, and Glu_491_ as described above.

**Figure 2:**
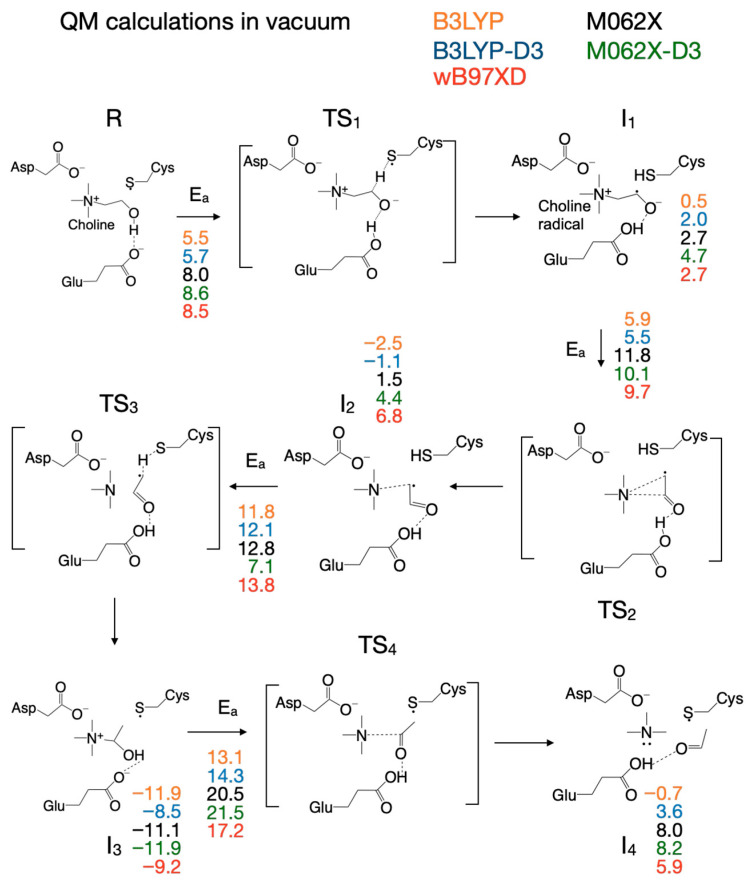
Scheme of the reaction mechanism from reactant (choline) to products (TMA and acetaldehyde), with intermediates and transition states calculated in vacuum using the DFT method. The reaction enthalpies relative to the reactant (in kcal/mol) and activation energies values listed for each individual chemical species along the reaction pathway are colored according to the DFT functional used as follows: B3LYP (orange), B3LYP-D3 (blue), M06-2X (black), M06-2X-D3 (green), and ωB97XD (red). The split-valence double-zeta basis set Def2-SVP was used.

To decide which DFT functional to use for QM/MM calculations, we refined the putative reaction mechanism obtained by QM cluster calculations at the HF/6-31G(d) level of ab initio theory by using three DFT functionals: (1) the B3LYP functional [59,60], an older and very popular DFT functional, used extensively for the last thirty years, together with its variant (B3LYP-D3) that includes dispersion interactions corrections [63,64]; (2) the M06-2X functional [61], together with its variant (M06-2X-D3) that includes dispersion interactions corrections [63,64]; and (3) the ωB97XD functional [62], which was designed to include dispersion interactions by default. The reaction mechanism calculated in vacuum with these three functionals (and two variants) using the split-valence double-zeta Def2-SVP basis set [65,66] is shown in Figure 2. The activation energies and reaction energies (in kcal/mol) relative to the reactant (R) were listed (color-coded) for each chemical species along the reaction pathway as follows: B3LYP (orange), B3LYP-D3 (blue), M06-2X (black), M06-2X-D3 (green), and ωB97XD (red). We note first that the reaction mechanism obtained with the DFT method in vacuum differs significantly from the reaction mechanism obtained with the HF method. When DFT is used, the first step in the reaction is a concerted transfer of the H atom from choline to the Cys_489_ radical and a proton from choline OH to the glutamate residue Glu_491_. This reaction step has an activation energy in the range of 5.5–8.5 kcal/mol, about four times lower than when the calculations are performed with the HF method, and is not the rate-limiting step. In the second reaction step, the C_2_-N bond is broken through a cyclic transition state (TS_2_, Figure 2), which requires an activation energy in the range of 5.5–11.8 kcal/mol, followed by the concerted transfer (TS_3_, Figure 2) of the H atom from Cys_489_ to the acetaldehyde radical and of the proton from the glutamic acid (Glu_491_) to acetaldehyde and the migration of TMA from C_2_ to C_1_ (i.e., C_2_-N bond is broken and a C_1_-N bond is formed), producing intermediate 3 (I_3_, Figure 2), which has choline substituted with carbinolamine compared to R. The activation energy in this third step is between 7.1 and 13.8 kcal/mol. Breaking the C_1_-N bond in carbinolamine is performed in step four (TS_4_, Figure 2) when the proton from the OH group of carbinolamine is transferred to the glutamate residue (Glu_491_) which requires between 13.1 and 21.5 kcal/mol depending on the DFT functional used. Compared to the HF method, which favors a TMA elimination mechanism, the DFT method favors a TMA migration mechanism that produces carbinolamine as an intermediate, a compound 8.5–11.9 kcal/mol more stable than choline itself within this surrogate enzyme environment, and has been claimed to be the end product of the choline-TMA lyase reaction by CutC in two recently published theoretical studies [50,51] (vide infra).

**Figure 3:**
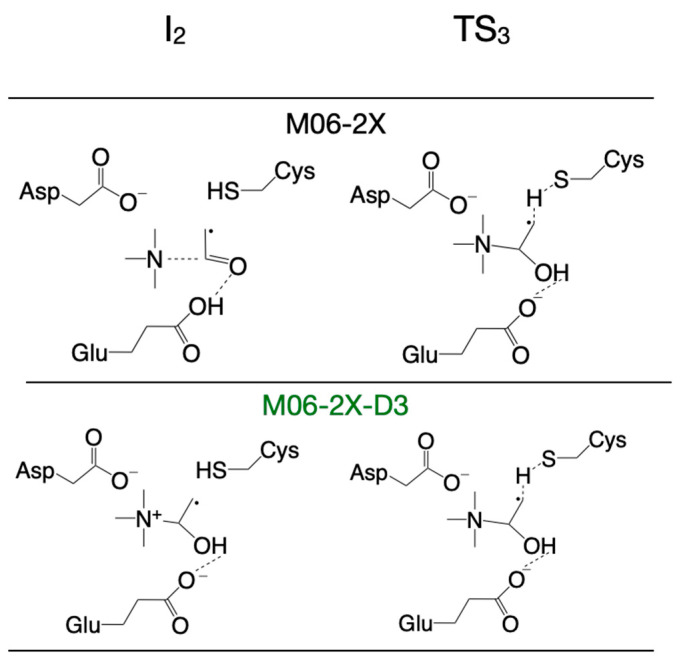
Differences in the reaction mechanism (e.g., intermediate I_2_ and transition state TS_3_) of choline lyase calculated in vacuum appear for DFT functionals M06-2X (black) and M06-2X-D3 (green). The basis set used is the split-valence double-zeta basis set Def2-SVP. The configuration of intermediate I_2_ changes when calculated with M06-2X-D3 in such a way that TMA of choline migrates from C_2_ to C_1_. The same reconfiguration is also observed for transition state TS_3_ when both M06-2X functionals (±D3) are used.

**Figure 4:**
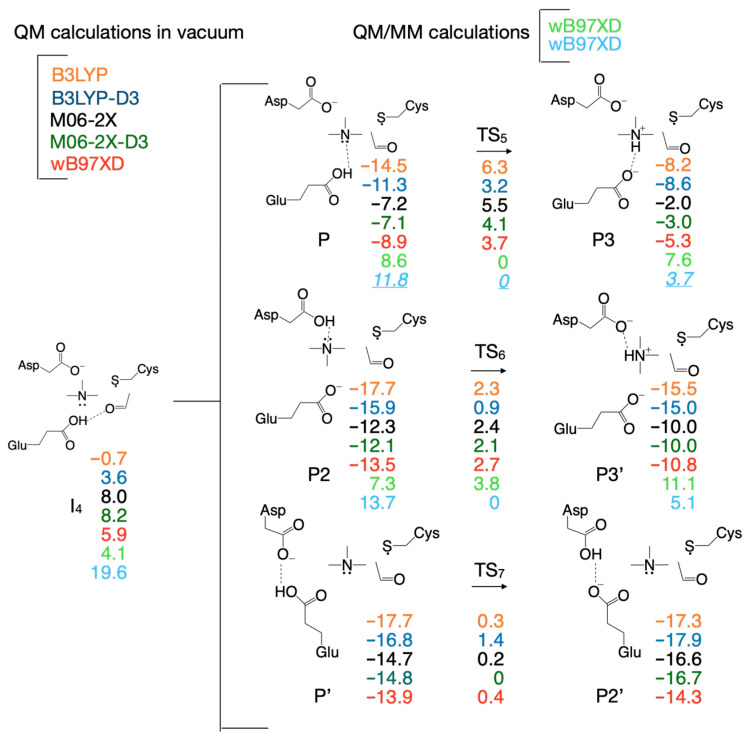
Following the breaking of choline into TMA and acetaldehyde, the proton from choline hydroxy group may end up (1) on TMA (P3 or P3’), making a H bond either with the glutamate residue (P3) or the aspartate residue (P3’); (2) on the glutamate residue (P or P’), making a H bond with TMA (P) or the aspartate residue (P’); or (3) on the aspartate residue (P2 or P2’), making a H bond with TMA (P2) or the glutamate residue (P2’). The cluster calculations were performed in vacuum with three different DFT functionals, B3LYP (orange), M06-2X (black), and ωB97XD (red), and with functionals augmented with dispersion interactions, B3LYP-D3 (blue) and M06-2X-D3 (green). The ωB97XD functional was designed to include dispersion interactions by default. The Figure also displays results for QM/MM calculations: ωB97XD/Amber force field without embedded MM partial atomic charges (light green) and with embedded MM partial atomic charges (light blue). The basis set used is Ahlrichs’ split-valence double-zeta basis set Def2-SVP. For the enzyme to restore its initial state and restart a new catalytic cycle, it seems necessary for TMA to leave the active site protonated (P3 or P3’). The underlined blue values were obtained by single point calculations on molecular geometries optimized without embedded MM partial atomic charges.

There are slight differences in the reaction mechanisms for intermediate I_2_ and transition state TS_3_ when the reaction pathway is calculated with these DFT functionals (Figure 3, Supplementary Table S1). For example, when the M06-2X functional (with/without dispersion correction, D3) is used for calculation, TS_3_ contains the carbinolamine radical already formed, compared to the configuration of TS_3_ calculated with the other two DFT functionals (B3LYP and ωB97XD) where TMA is not bonded to the acetaldehyde radical (Figure 3, right); the configuration of I_2_ is different (Figure 3, left) when M06-2X is used with or without dispersion correction. While the reaction mechanism scheme in Figure 2 shows TMA and acetaldehyde fully formed in step four, we note that the hydroxy group proton from carbinolamine is now bonded to Glu_491_ (intermediate I_4_, Figure 2). For the enzyme to revert to its initial state and restart a new catalytic cycle, it makes sense to assume that the proton will ultimately be transferred to TMA (a stronger base than water), which should leave the enzyme active site in protonated form. Considering that the proton from the OH group of choline may end up either on Glu_491_ or Asp_216_ (perhaps via Thr_502_), Figure 4 shows possible proton transfer reactions between glutamic acid (Glu_491_) and TMA (TS_5_), aspartic acid (Asp_216_) and TMA (TS_6_), and between glutamic acid (Glu_491_) and aspartic acid (Asp_216_) (TS_7_) that may constitute the last step in the reaction mechanism. All these proton transfer reactions are significantly exothermic. The activation energies and energies relative to the reactant are listed (color-coded) for each DFT functional (Figure 4) used as in Figure 2. The values in light green and light blue are from QM/MM calculations (vide infra). The activation energies are rather small, and the proton transfer reaction slightly favors having the proton attached to an acidic amino acid instead of TMA (Figure 4).

Keeping in mind our decision to ultimately use the QM/MM ONIOM method [71] for exploring the reaction mechanism in the enzyme environment, and the preference of including dispersion interactions into calculations, we found out that only the ωB97XD functional [62] can accommodate this combination within the ONIOM method [71] implemented in the Gaussian16 program [58]. Therefore, we selected the ωB97XD functional with the split-valence double-zeta basis set Def2-SVP [65,66] to proceed with the QM/MM calculations.

### 3.3. Setup of the QM/MM System for Reaction Mechanism Calculations Within Enzyme Environment

**Figure 5:**
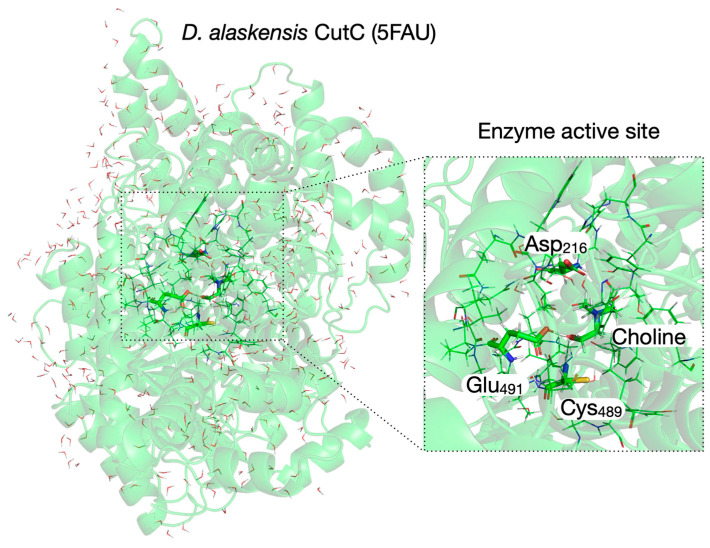
Setup of the QM/MM system used to investigate the reaction mechanism of choline lyase by the gut microbial enzyme *D. alaskensis* CutC (PDB id: 5FAU). Active site residues in the QM subsystem (CholineActiveSite-1) are drawn with sticks (carbon = green, oxygen = red, nitrogen = blue, hydrogen = white), and the residues in the MM subsystem, which are allowed to move during geometry optimization, transition state search, or internal reaction coordinate (IRC) calculations (within 3.5 Å from the QM subsystem), are drawn with lines. The backbone of CutC is shown as a semi-transparent ribbon, and crystallization water is also shown with lines. The configuration of the residues in the active site and choline substrate corresponds to the reactant.

For QM/MM calculations, we included in the QM/MM system the entire *D. alaskensis* CutC monomer and the crystallization water present in the PDB entry 5FAU [36]. The QM/MM system is shown in Figure 5 (left panel). The QM/MM system has the CholineActiveSite-1 residues (drawn with sticks) described by QM and the rest of the enzyme residues (shown as protein backbone ribbon) and water molecules (drawn with lines) described by MM (using the Amber force field [72] as implemented in the ONIOM method [71] of the Gaussian16 program [58]). In the QM subsystem, the “terminal” amide and carbonyl groups of the amino acid backbone were replaced by hydrogen link atoms, making a C_α_ methyl group. Amino acid residues and water molecules within 3.5 Å of CholineActiveSite-1 (drawn with lines, Figure 5, right panel; see Methods) were allowed to move during geometry optimization and transition state search.

**Figure 6:**
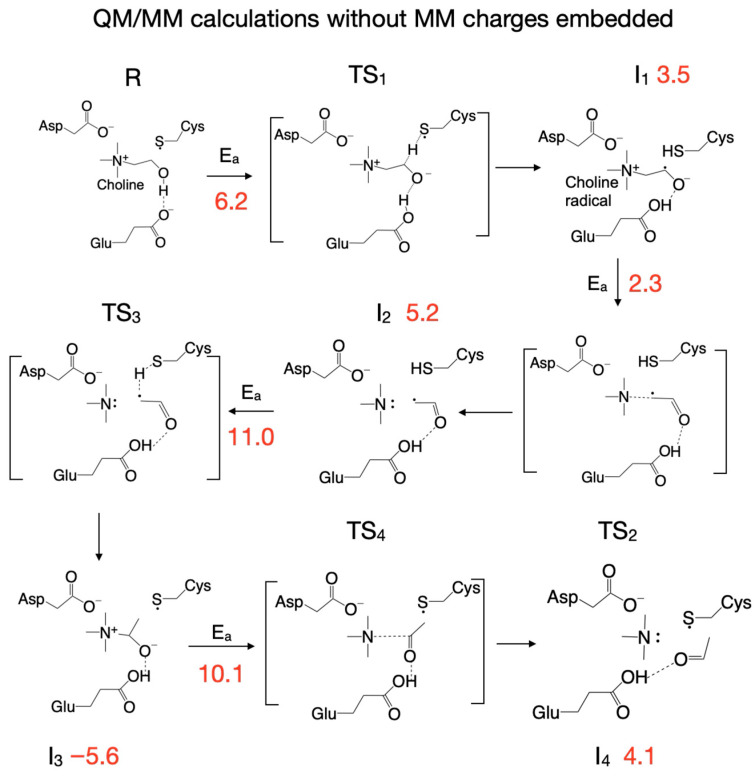
Scheme of the reaction mechanism from reactant to product with intermediates and transition states calculated by the QM/MM method without embedding into the QM region the partial atomic charges of the atoms described by molecular mechanics (MM). The calculations were performed using the ONIOM method with the ωB97XD functional (red values) and the split-valence double-zeta Def2-SVP basis set. The Amber force field was used to describe the interaction of atoms in the MM region. The activation energies and reaction energies relative to the reactant (in kcal/mol) are listed for each individual chemical species along the reaction pathway.

The enzyme environment provides both a restricting (steric) and a polarization environment for the enzymatic reaction. The steric (restricting, cage) effect is due to the protein’s compact structure that defines an active site cavity with a well-defined shape tailored for the substrate, which has strategically placed amino acid residues that can activate the substrate and start the reaction. This cage effect also contributes to reaction energetics by diminishing the substrate’s entropy due to its translational and rotational/librational motion. In addition to the steric effect, the enzyme and solvent have a polarizing effect on the wave function describing the QM subsystem, through an external inhomogeneous and anisotropic electric field created by the partial atomic charges of the atoms of the enzyme (and solvent molecules) described by MM. The implementation of the ONIOM method [71] for the QM/MM calculations in the Gaussian16 program [58] allows us to separate these two enzymatic effects (steric and electronic) by the use of two calculation scenarios. (1) In the first scenario, the QM/MM calculations are performed without including the partial atomic charges of the atoms described by MM into the wave function that describes the QM subsystem. This leads to a reaction mechanism in which only the steric (cage) effect of the enzyme’s 3D structure on the reaction is taken into consideration. (2) In the second scenario, the QM/MM calculations are performed by including the MM partial atomic charges into the wave function that describes the QM subsystem. In this latter scenario, the electric field due to the partial atomic charges of atoms described by MM distorts the wave function of the QM subsystem to some extent, affecting both the energetics of the reaction and the geometrical configuration of the chemical species (reactant, product, intermediates, transition states) along the reaction path (Figure 6). When using the ONIOM method for QM/MM, the calculations using the second scenario are slower and less stable, especially when transition states are searched; however, the polarizing effect of the electric field of the enzyme (and solvent) on the active site can be significant and may alter the reaction mechanism. Therefore, we decided to first determine the reaction mechanism in the enzyme environment by accounting for the steric effect alone (i.e., without including the MM partial atomic charges in calculations), and then, in the next step, we refined the reaction mechanism by including the MM partial atomic charges, thus accounting for the combination of both steric and polarization effects due to enzyme and solvent environment.

### 3.4. Reaction Mechanism from Exploring the PES with QM/MM Calculations Performed Without Including MM Partial Atomic Charges into the QM Subsystem

The reaction mechanism obtained by performing QM/MM calculations without embedding the MM partial atomic charges into the QM subsystem is shown in Figure 6. Like in the cluster vacuum calculations using the ωB97XD functional with the split-valence double-zeta Def2-SVP basis set, the first step in the reaction is the concerted transfer of a H atom to the Cys_489_ radical and the proton from choline’s alcoholic group to Glu_491_, which requires an activation energy of 6.2 kcal/mol (compared to 8.5 kcal/mol in vacuum; Figure 2). Note that the proton transfer seems to precede the H atom transfer because, in TS_1_ (Figure 6), the proton is already bonded to Glu_491_. In the second step the C_2_-N bond in the choline radical is broken (TS_2_, Figure 6), requiring an activation energy of 2.3 kcal/mol (compared to 9.7 kcal/mol in vacuum, Figure 2), and in the third step the H atom is transferred back from Cys_489_ to the acetaldehyde radical (TS_3_, Figure 6) followed by TMA migration from C_2_ to C_1_ (through the breaking of the C_2_-N bond and the formation of the C_1_-N bond) in intermediate 3 (I_3_, Figure 6). The activation energy for this reaction step is 11 kcal/mol (compared to 13.8 kcal/mol in vacuum, Figure 2). We note that compared to vacuum calculation for step three, the proton of the glutamic acid (Glu_491_) is not transferred back to form carbinolamine (I_3_, Figure 2) but rather remains bonded to Glu_491_ (I_3_, Figure 6). Breaking the C_1_-N bond in step four requires an activation energy of 10.1 kcal/mol (compared to 17.2 kcal/mol in vacuum). The rate-limiting step seems to be the H atom transfer back from Cys_489_ to the acetaldehyde radical; however, breaking the C_1_-N bond seems to require almost the same amount of activation energy (11. 0 versus 10.1 kcal/mol, Figure 6). The main difference between the cluster vacuum and the QM/MM calculation (without MM partial atomic charges embedded) seems to be the lowering of the activation energies of the reaction steps along the reaction path and the absence of the fully formed carbinolamine intermediate (I_3_). However, I_3_ obtained in cluster vacuum calculations (Figure 2) differs from I_3_ obtained in QM/MM calculations (Figure 6) only by the location of choline hydroxyl proton. I_3_ obtained in cluster vacuum calculations is 13.3 kcal/mol more stable than the one obtained in QM/MM calculations. The attempt to transfer the hydroxyl proton from Glu_491_ to TMA is enthalpically favorable (−1 kcal/mol), but the transfer from Asp_216_ to TMA is unfavorable (+3.8 kcal/mol) (light green values in Figure 4).

Exploring the PES for choline-TMA lyase within the enzyme environment using the ONIOM method (Gaussian16 program) turned out to be more difficult than when using cluster vacuum calculations. We were able to locate TS_1_ and TS_3_ by using the transition state search capabilities of the program, while the rest of the transition states (TS_2_, TS_4_, TS_5_, TS_6_) were located by following a reaction coordinate (usually the distance between two atoms) with constrained optimizations. For example, for the second step of the reaction we located the transition state for breaking the C_2_-N bond in the choline radical (TS_2_) by constraining the distance between C_2_ and N atoms and performing constrained geometry optimization. The profile of the PES obtained for this reaction coordinate (Supplementary Figure S4) indicates that there are two transition states (TS_2_ and TS_2_’) with activation energies of 2.3 and 0.8 kcal/mol, corresponding to the distance between C_2_ and N of 1.875 and 2.23 Å, respectively. The bottom of Supplementary Figure S4 displays the calculated structures of intermediates I_1_ and I_2_ and transition states TS_2_ and TS_2_’. We identified transition state 4 (TS_4_) for breaking the C_1_-N bond in carbinolamine in an analogous way. We performed constrained geometry optimization while fixing the distance between C_1_ and N atoms. The profile of the PES obtained for this reaction coordinate (Supplementary Figure S5) shows that transition state TS_4_ has an activation energy of 10.1 kcal/mol and is located on the PES at the distance between C_1_ and N of 3.13 Å, very close to intermediate 4 (I_4_). The bottom of Supplementary Figure S5 displays the calculated structures of intermediates I_3_ and I_4_ and transition state TS_4_.

### 3.5. Reaction Mechanism from Exploring the PES with QM/MM Calculations Performed by Including the MM Partial Atomic Charges into the QM Subsystem

**Figure 7:**
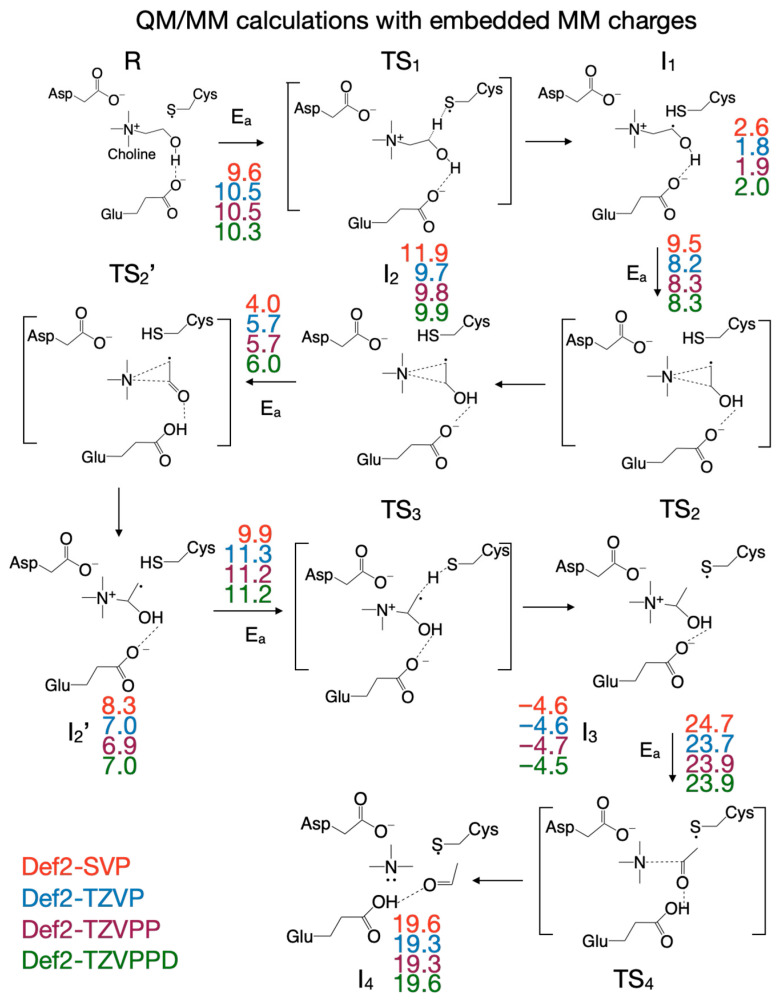
Scheme of the reaction mechanism from reactant to product with intermediates and transition states calculated by embedding the partial atomic charges of atoms described by molecular mechanics (MM) into the QM region. The calculations were performed using the ONIOM method with the ωB97XD functional and the split-valence double-zeta Def2-SVP basis set (red values). The PES was refined by also using more sophisticated basis sets: split-valence triple-zeta Def2-TZVP (blue values) and basis sets which contain additional polarization (Def2-TZVPP, brown values) and diffusion functions (Def2-TZVPPD, green values). The Amber force field was used to describe the interaction of atoms described by MM.

To capture the entire effect (steric and electronic) of protein environment and crystallization water on the reaction mechanism, we next included the partial atomic charges of all atoms described by MM into the QM subsystem. The cloud of MM partial atomic charges forms an inhomogeneous anisotropic electric field that distorts the wave function of the QM subsystem (CholineActiveSite-1), thus incorporating the full effect of the enzyme and crystallization water into the energetics of the reaction mechanism. Therefore, we used the PES for the reaction mechanism obtained from QM/MM calculations, performed without embedding the MM partial atomic charges as the starting point for exploring the PES for the reaction mechanism when the MM partial atomic charges were embedded into the QM subsystem, because we assumed that the geometries of the intermediates and transition states obtained by exploring the PES, without including the MM partial atomic charges, are the best guesses for the geometries of the intermediates and transition states to be found on the PES with the MM partial atomic charges included. Our assumption was based on the idea that the QM/MM calculations performed without embedding the MM partial atomic charges already incorporate into the PES the restricting (cage) effect that the enzyme’s 3D structure has on the reaction pathway. We found significant differences between the reaction mechanism obtained without and with the MM partial atomic charges embedded. When the MM partial atomic charges are included, the first step in the reaction is the H atom transfer from choline to the Cys_489_ radical, without also transferring the hydroxyl proton of choline to Glu_491_ (Figure 7). The activation energy of this step is 9.6 kcal/mol (compared to 6.2 kcal/mol for the step without the MM partial atomic charges embedded). The second step of the reaction (breaking the C_2_-N bond) leads to an intermediate (I_2_, Figure 7) which resembles transition state 2 (TS_2_, activation energy 9.5 kcal/mol). In the third step of the reaction, the proton from the hydroxyl group of choline is temporarily transferred to Glu_491_, leading to transition state 2’ (TS_2_’, Figure 7, activation energy of 4.0 kcal/mol). Intermediate 2’ (I_2_’, Figure 7), formed in step three, contains the carbinolamine radical. TS_2_’ and I_2_’ have no correspondents on the PES of the reaction calculated without MM partial atomic charges embedded, and they were labeled with prime to make it easier to compare the reaction mechanisms obtained with the two QM/MM computational protocols (i.e., with/without embedding MM partial atomic charges) available in the ONIOM method. In step four of the reaction, the H atom is transferred back from Cys_489_ to the carbinolamine radical through transition state 3 (TS_3_, Figure 7), producing carbinolamine which is 4.6 kcal/mol more stable than choline in this environment. Finally, in the fifth step the C_1_-N bond is broken, requiring an activation energy of 24.7 kcal/mol. Surprisingly, intermediate 4 (I_4_, Figure 7) is 15.5 kcal/mol less stable than the one obtained without embedding MM partial atomic charges.

Some of the transition states (TS_2_, TS_2_’, TS_4_) were located manually through constrained optimization following a specific reaction coordinate (C_2_-N distance for TS_2_ and TS_2_’, and C_1_-N distance for TS_4_). The second step of the reaction constitutes the beginning of the TMA migration by breaking the C_2_-N in the choline radical (I_1_, Supplementary Figure S6), which leads to an intermediate (I_2_, Supplementary Figure S6) that contains an adduct between TMA and an enol radical in which the C_1_-N and C_2_-N distances are almost equal (2.876 Å and 2.819 Å), C_1_-C_2_ distance is 1.351 Å (a typical C-C double bond), and the distance between the O and H atoms in the hydroxyl group is 1.047 Å. The enol radical is flat, confirming that C_1_ and C_2_ have sp^2^ hybridization. Intermediate I_2_ was confirmed as a minimum energy structure (no imaginary frequency) on the PES. The transition state TS_2_ leading to I_2_ was found manually by constrained optimizations using the C_2_-N distance as a reaction coordinate (Supplementary Figure S6, top-left panel), has an activation energy of 9.5 kcal/mol, and is very similar geometrically with I_2_, with C_1_-N and C_2_-N distances of 2.824 Å and 2.751 Å, respectively; the C_1_-C_2_ distance is 1.352 Å, and the distance between the O and H atoms in the hydroxyl group is 1.05 Å. While the hydroxyl group of the choline radical seems to remain intact during this reaction step, the PES exploration along the C_2_-N reaction coordinate reveals something surprising: the proton is transferred back and forth to Glu_491_ before reaching the transition state as shown in the top-right panel of Supplementary Figure S6. The forward proton transfer (to Glu_491_) starts when the C_2_-N distance is ~2.0 Å, and the transfer back starts when the C_2_-N distance is ~2.4 Å. This unexpected back-and-forth proton transfer supports the hypothesis that Glu_491_ acts as a weak base at some point during the reaction and is not there only as a hydrogen bond acceptor to help orient/polarize choline into the active site. The optimized geometries of I_1_, TS_2_, and I_2_ are shown at the bottom of Supplementary Figure S6.

In the third step of the reaction (Supplementary Figure S7), I_2_ continues the TMA migration by forming a C_1_-N bond leading to the carbinolamine radical (I_2_’) through transition state TS_2_’, which has an activation energy of 4.0 kcal/mol and is found at a C_1_-N distance of 2.101 Å. (Supplementary Figure S7, top-left panel). TS_2_’ geometrically resembles I_2_, with C_1_-N and C_2_-N distances of 2.101 Å and 2.700 Å; the C_1_-C_2_ distance is 1.442 Å (greater than a typical C-C double bond), the C_1_-O distance is 1.267 Å (typical carbonyl bond), the distance between the O and H atoms in the hydroxyl group is 1.347 Å, and the distance between the proton and Glu_491_ is 1.085 Å. The acetaldehyde radical is not planar, indicating that C_1_ and C_2_ have hybridization between sp^2^ and sp^3^. The top-right panel of Supplementary Figure S7 shows that the hydroxylic proton of the enol radical (I_2_) is transferred to Glu_491_ before reaching TS_2_’ and then transferred back when the carbinolamine radical (I_2_’) is formed. I_2_’ is only 3.6 kcal/mol more stable than I_2_; the geometry-optimized structures involved in the third reaction step are shown at the bottom of Supplementary Figure S7.

After the H atom is transferred back from Cys_489_ to the carbinolamine radical (reaction step four, Figure 7), the C_1_-N bond is broken in the fifth step of the reaction (Supplementary Figure S8) to produce TMA and acetaldehyde. The transition state (TS_4_) for this reaction step was also located manually by constrained geometry optimizations along the reaction coordinate defined by the C_1_-N distance. Reaching the transition state requires a large activation energy (24.7 kcal/mol), making this reaction step the rate-limiting step for the conversion of choline to TMA and acetaldehyde within the enzyme environment. The transition state TS_4_ was located at a C_1_-N distance of 2.901 Å (Supplementary Figure S8, top-left panel). The hydroxylic proton of carbinolamine is transferred to Glu_491_ before reaching the transition state (Supplementary Figure S8, top-right panel). If TMA and acetaldehyde (I_4_) are the final products of the enzymatic reaction, then Glu_491_ is needed, at least temporarily, to host the hydroxylic proton (vide supra), confirming that it plays the role of a weak base in this reaction mechanism. Intermediate I_3_ is 24.2 kcal/mol more stable than I_4_; the geometry-optimized structures involved in the fifth reaction step are shown at the bottom of Supplementary Figure S8.

In an attempt to refine the PES for the choline-TMA lyase reaction obtained with this last computational protocol, we performed additional single-point calculations using more sophisticated split-valence triple-zeta basis sets (Def2-TZVP) that include additional polarization (Def2-TZVPP) and diffusion (Def2-TZVPPD) basis functions. These refinements of the PES (shown in Figure 7, color-coded) do not change the reaction mechanism and do not affect the reaction energetics significantly.

### 3.6. Steric and Electronic Effects of the Enzyme Environment on the Choline-TMA Lyase Reaction Mechanism

When investigating the reaction mechanism in the enzyme environment through QM/MM calculations, it is computationally efficient to map first the reaction mechanism in vacuum using a QM system composed of the substrate and chemical surrogates of the amino acids within the enzyme active site that directly participate in the reaction or assist in breaking/forming chemical bonds that lead to the product. The QM cluster exploration of the PES in vacuum produces transition states and intermediates that can be used as starting configurations for exploring the PES in the enzyme environment, thus saving significant computational resources. In addition to generating these putative configurations for the QM/MM calculations, the vacuum cluster calculations help to better understand the effect of the enzyme environment on the reaction. We can think of this effect as being a combination of steric (cage) and electronic (polarization) effects, the former being due to the physical constraints that the compact structure of the enzyme imposes on the reactant, transition states, and intermediates by significantly reducing its translational/rotational/librational motion, and the latter effect being due to the electric field of the partial charges of the enzyme atoms described by MM that distorts the wave function of the QM subsystem.

**Figure 8:**
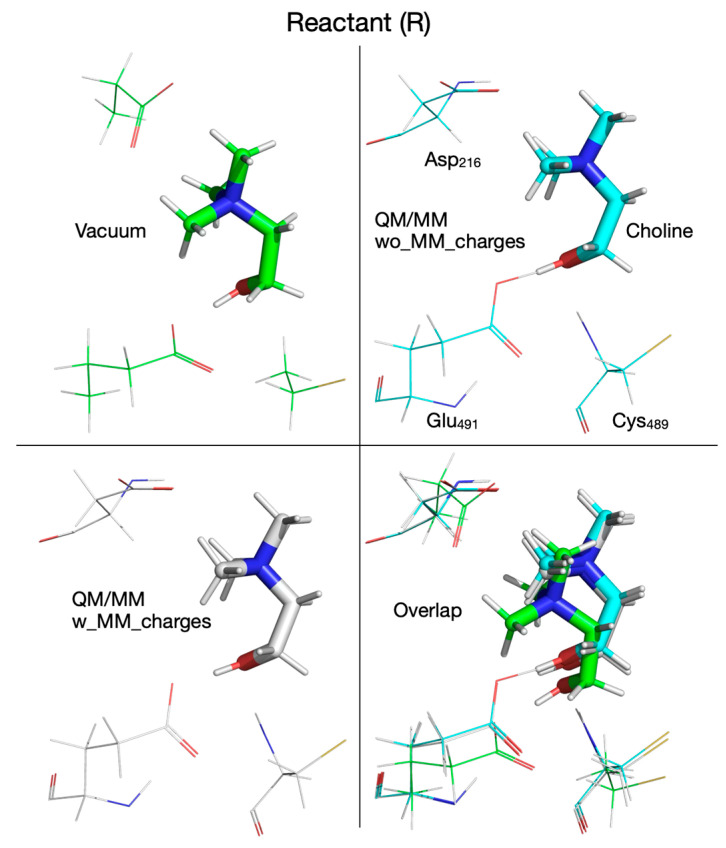
Comparison of reactant (R) configurations obtained by using the ωB97XD functional and the split-valence double-zeta Def2-SVP basis set in vacuum (**top left**), in the enzyme environment without embedding the MM partial atomic charges in the QM subsystem (**top-right**), and by embedding the MM partial atomic charges in the QM subsystem (**bottom left**). The overlap of the R configurations obtained by the three different calculation setups is shown in the (**bottom-right**) panel (carbon = green/cyan/grey, oxygen = red, nitrogen = blue, hydrogen = white).

**Figure 9:**
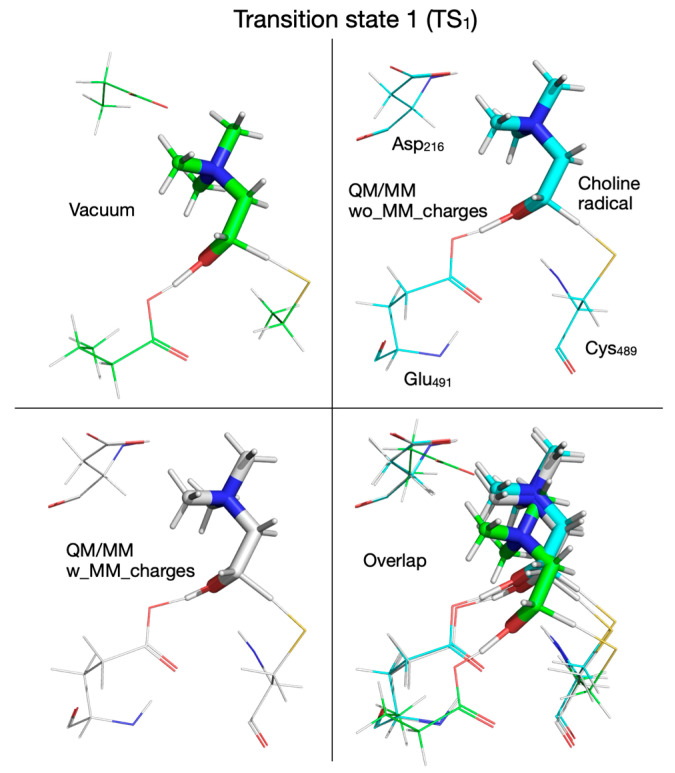
Comparison of transition state 1 (TS_1_) configurations obtained by using the ωB97XD functional and the split-valence double-zeta Def2-SVP basis set in vacuum (**top left**), in the enzyme environment without embedding the MM partial atomic charges in the QM subsystem (**top right**), and by embedding the MM partial atomic charges in the QM subsystem (**bottom left**). The overlap of the TS_1_ configurations obtained by the three different calculation setups is shown in the (**bottom-right**) panel (carbon = green/cyan/grey, oxygen = red, nitrogen = blue, hydrogen = white).

**Figure 10:**
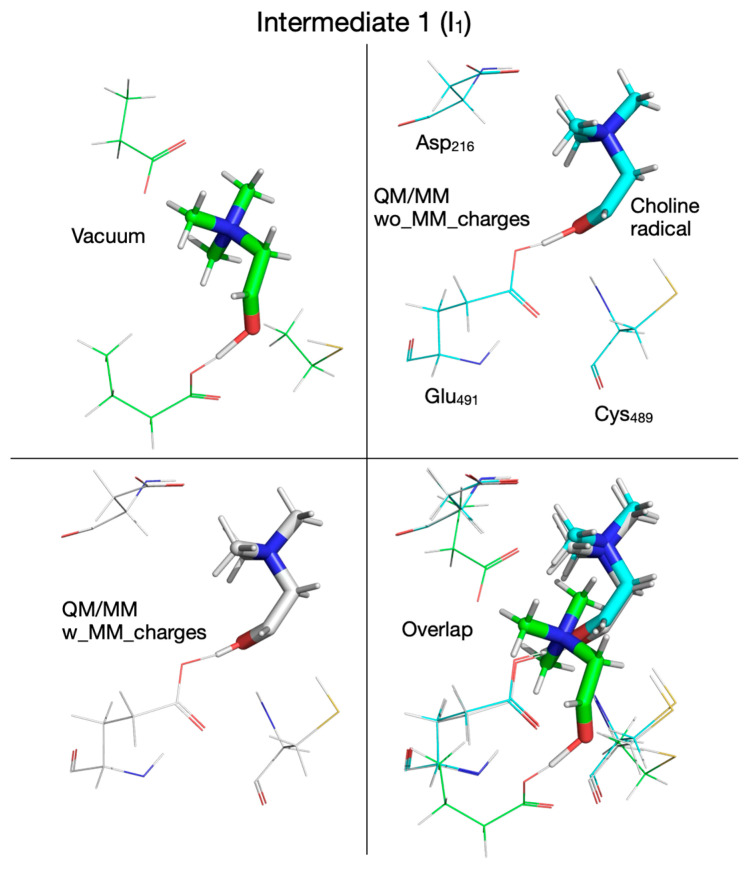
Comparison of intermediate 1 (I_1_) configurations obtained by using the ωB97XD functional and the split-valence double-zeta Def2SVP basis set in vacuum (**top left**), in the enzyme environment without embedding the MM partial atomic charges in the QM subsystem (**top right**), and by embedding the MM partial atomic charges in the QM subsystem (**bottom left**). The overlap of the I_1_ configurations obtained by the three different calculation setups is shown in the (**bottom-right**) panel (carbon = green/cyan/grey, oxygen = red, nitrogen = blue, hydrogen = white).

**Figure 11:**
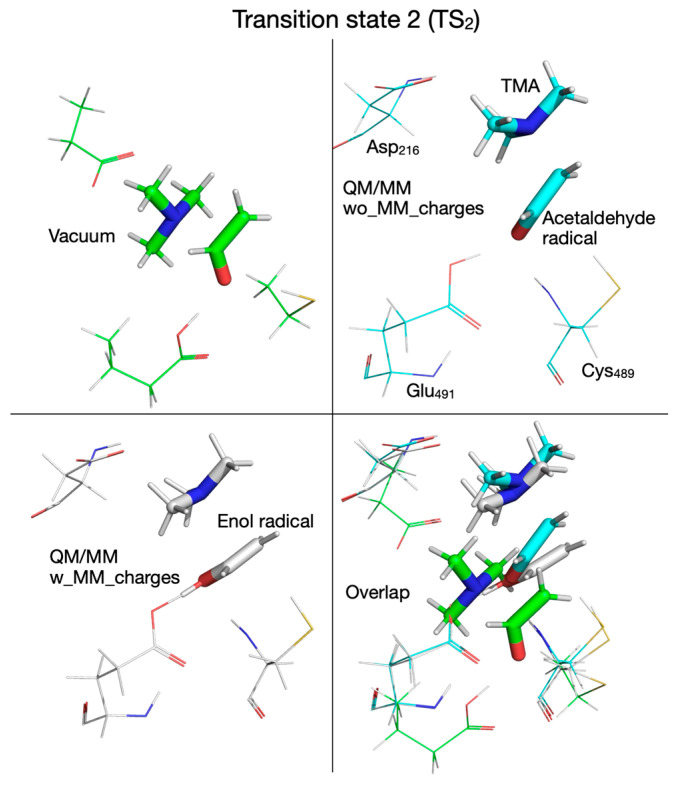
Comparison of transition state 2 (TS_2_) configurations obtained by using the ωB97XD functional and the split-valence double-zeta Def2-SVP basis set in vacuum (**top left**), in the enzyme environment without embedding the MM partial atomic charges in the QM subsystem (**top right**), and by embedding the MM partial atomic charges in the QM subsystem (**bottom left**). The overlap of the TS_2_ configurations obtained by the three different calculation setups is shown in the (**bottom-right**) panel (carbon = green/cyan/grey, oxygen = red, nitrogen = blue, hydrogen = white).

**Figure 12:**
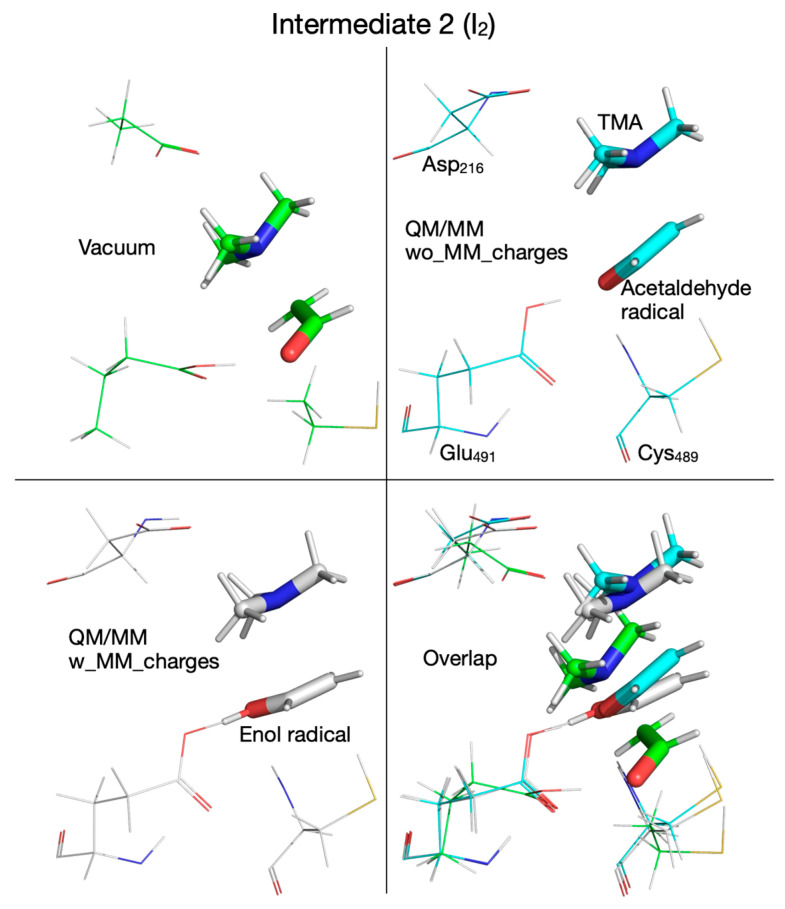
Comparison of intermediate 2 (I_2_) configurations obtained by using the ωB97XD functional and the split-valence double-zeta Def2-SVP basis set in vacuum (**top left**), in the enzyme environment without embedding the MM partial atomic charges in the QM subsystem (**top right**), and by embedding the MM partial atomic charges in the QM subsystem (**bottom left**). The overlap of the I_2_ configurations obtained by the three different calculation setups is shown in the (**bottom-right**) panel (carbon = green/cyan/grey, oxygen = red, nitrogen = blue, hydrogen = white).

**Figure 13:**
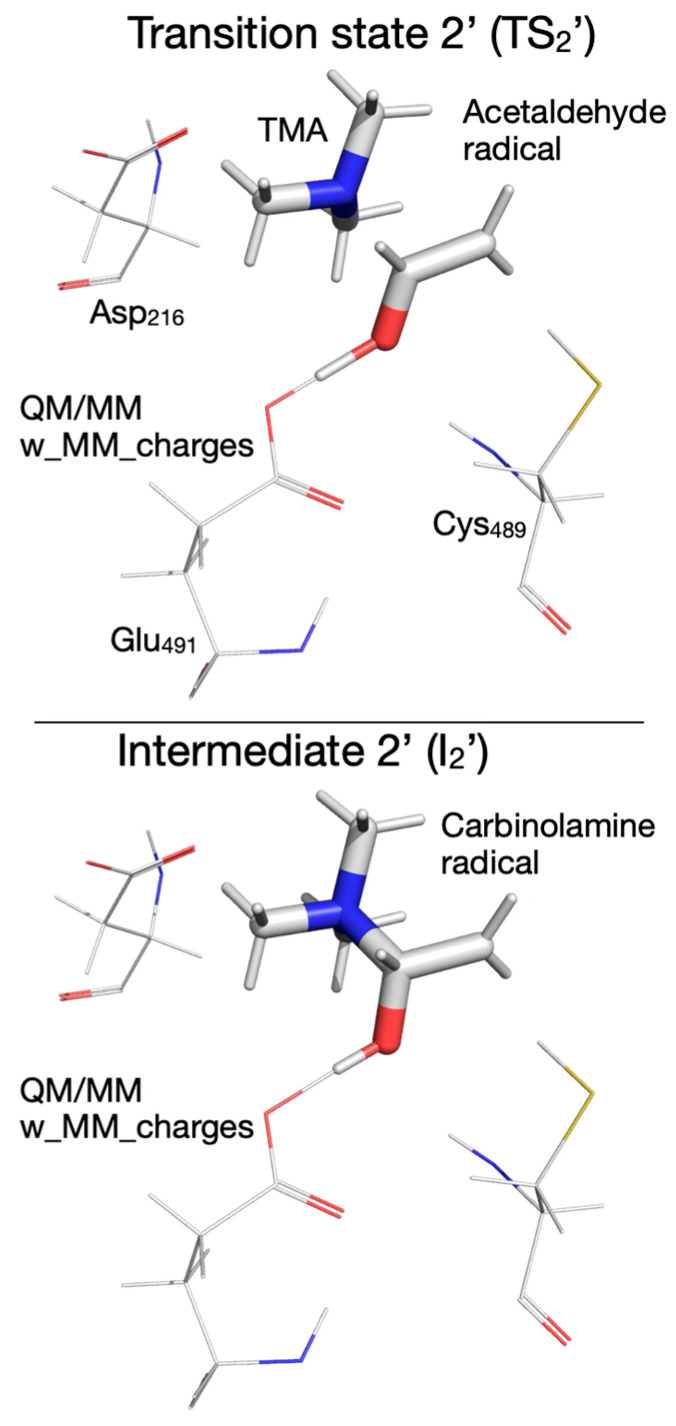
Configurations of the transition state 2’ (TS_2_’) (**top**) and intermediate 2’ (I_2_’) (**bottom**) obtained by using the ωB97XD functional and the split-valence double-zeta Def2-SVP basis set and by embedding the MM partial atomic charges in the QM subsystem (carbon = grey, oxygen = red, nitrogen = blue, hydrogen = white).

**Figure 14:**
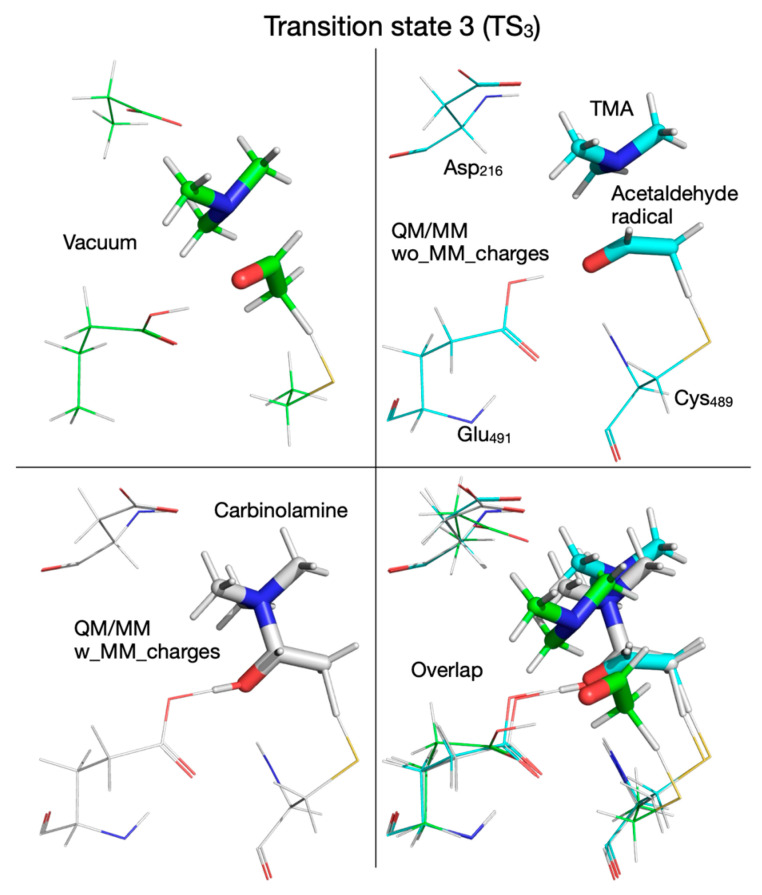
Comparison of transition state 3 (TS_3_) configurations obtained by using the ωB97XD functional and the split-valence double-zeta Def2-SVP basis set in vacuum (**top left**), in the enzyme environment without embedding the MM partial atomic charges in the QM subsystem (**top right**), and by embedding the MM partial atomic charges in the QM subsystem (**bottom left**). The overlap of the TS_3_ configurations obtained by the three different calculation setups is shown in the (**bottom-right**) panel (carbon = green/cyan/grey, oxygen = red, nitrogen = blue, hydrogen = white).

**Figure 15:**
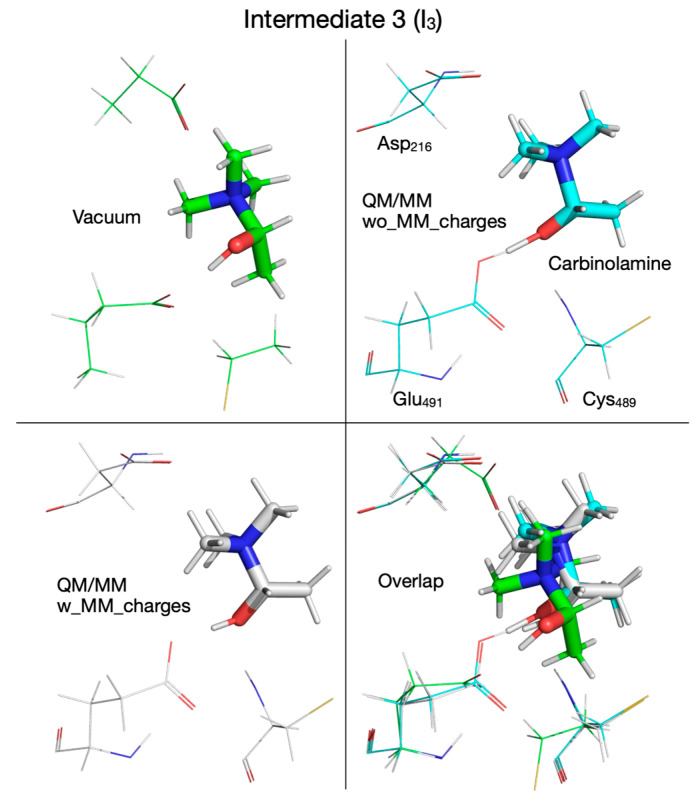
Comparison of intermediate 3 (I_3_) configurations obtained by using the ωB97XD functional and the split-valence double-zeta Def2-SVP basis set in vacuum (**top left**), in the enzyme environment without embedding the MM partial atomic charges in the QM subsystem (**top right**), and by embedding the MM partial atomic charges in the QM subsystem (**bottom left**). The overlap of the I_3_ configurations obtained by the three different calculation setups is shown in the (**bottom-right**) panel (carbon = green/cyan/grey, oxygen = red, nitrogen = blue, hydrogen = white).

**Figure 16:**
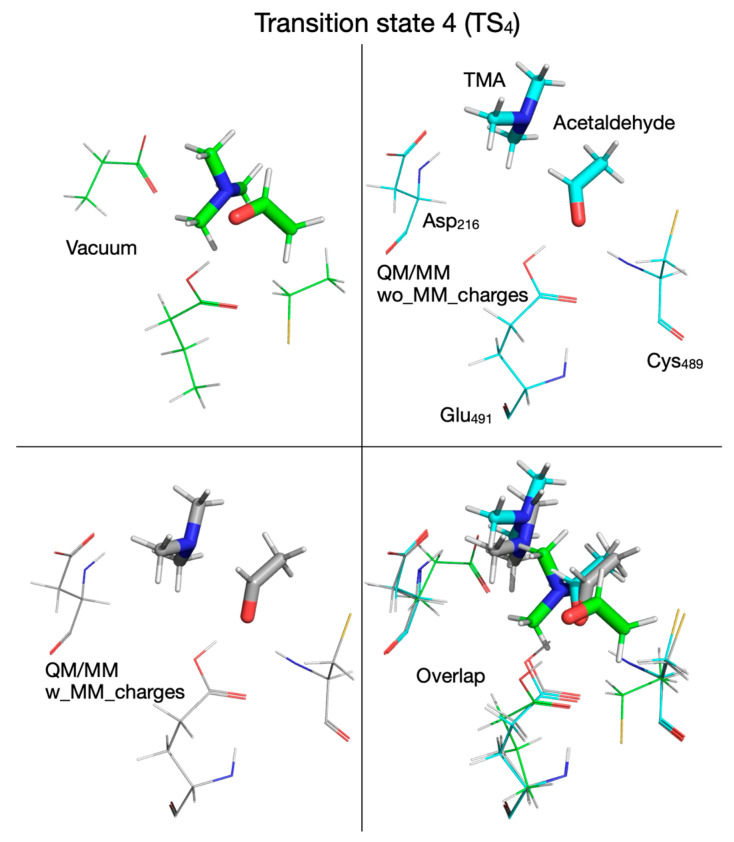
Comparison of transition state 4 (TS_4_) configurations obtained by using the ωB97XD functional and the split-valence double-zeta Def2-SVP basis set in vacuum (**top left**), in the enzyme environment without embedding the MM partial atomic charges in the QM subsystem (**top right**), and by embedding the MM partial atomic charges in the QM subsystem (**bottom left**). The overlap of the TS_4_ configurations obtained by the three different calculation setups is shown in the (**bottom-right**) panel (carbon = green/cyan/grey, oxygen = red, nitrogen = blue, hydrogen = white).

**Figure 17:**
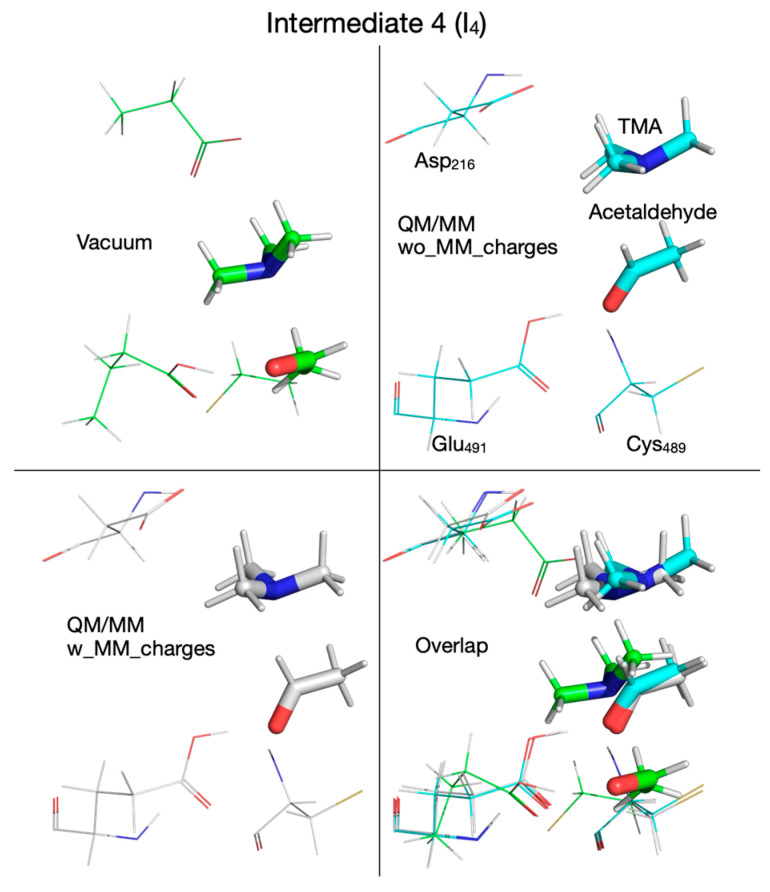
Comparison of intermediate 4 (I_4_) configurations obtained by using the ωB97XD functional and the split-valence double-zeta Def2-SVP basis set in vacuum (**top left**), in the enzyme environment without embedding the MM partial atomic charges in the QM subsystem (**top right**), and by embedding the MM partial atomic charges in the QM subsystem (**bottom left**). The overlap of the I_4_ configurations obtained by the three different calculation setups is shown in the (**bottom-right**) panel (carbon = green/cyan/grey, oxygen = red, nitrogen = blue, hydrogen = white).

**Figure 18:**
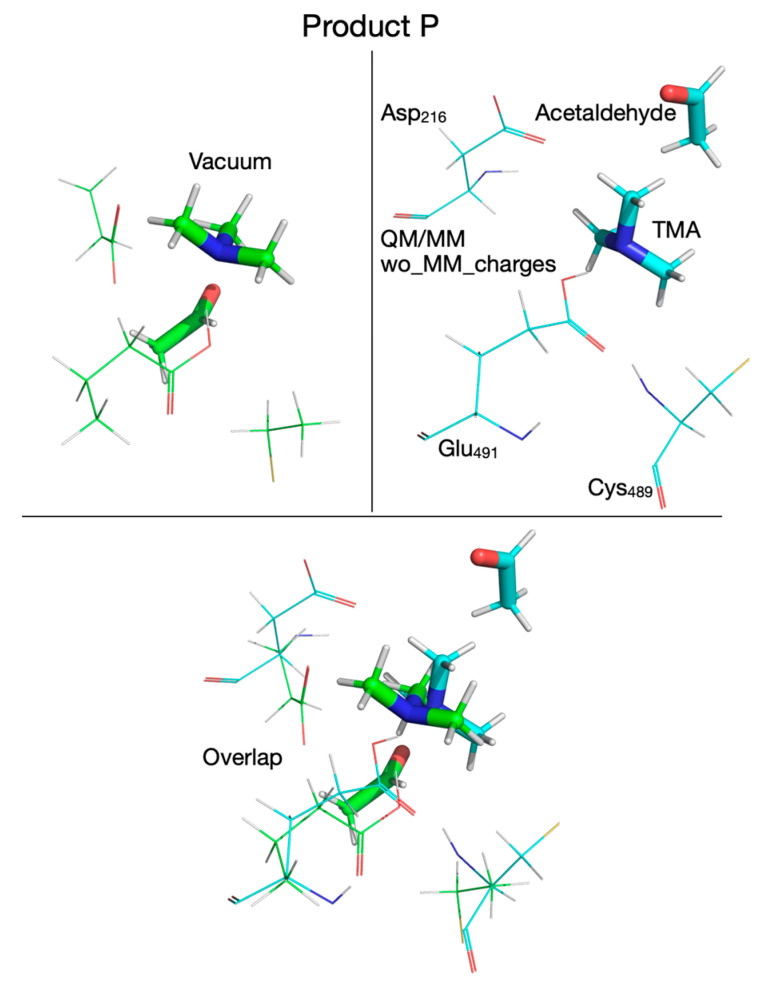
Comparison of product P configurations obtained by using the ωB97XD functional and the split-valence double-zeta Def2-SVP basis set in vacuum (**top left**) and in the enzyme environment without embedding the MM partial atomic charges in the QM subsystem (**top right**). The overlap of P configurations obtained by the two different calculation setups is shown in the (**bottom**) panel (carbon = green/cyan, oxygen = red, nitrogen = blue, hydrogen = white).

**Figure 19:**
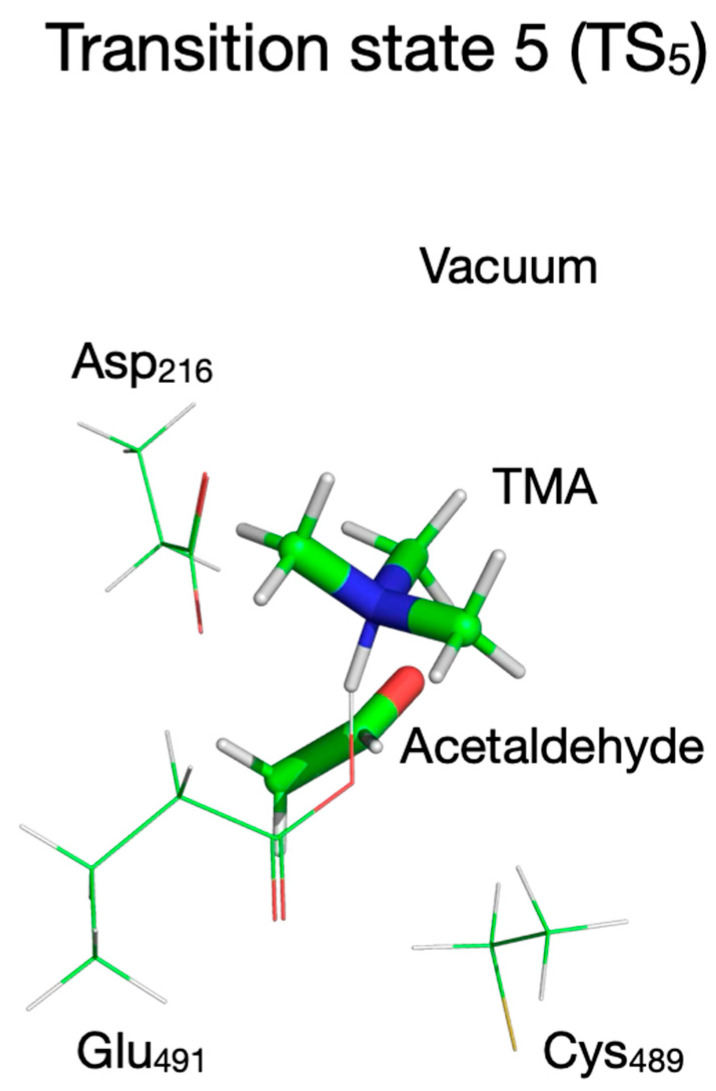
Transition state (TS_5_) for proton transfer from Glu_491_ to TMA obtained by using the ωB97XD functional and the split-valence double-zeta Def2-SVP basis set in vacuum (carbon = green, oxygen = red, nitrogen = blue, hydrogen = white).

**Figure 20:**
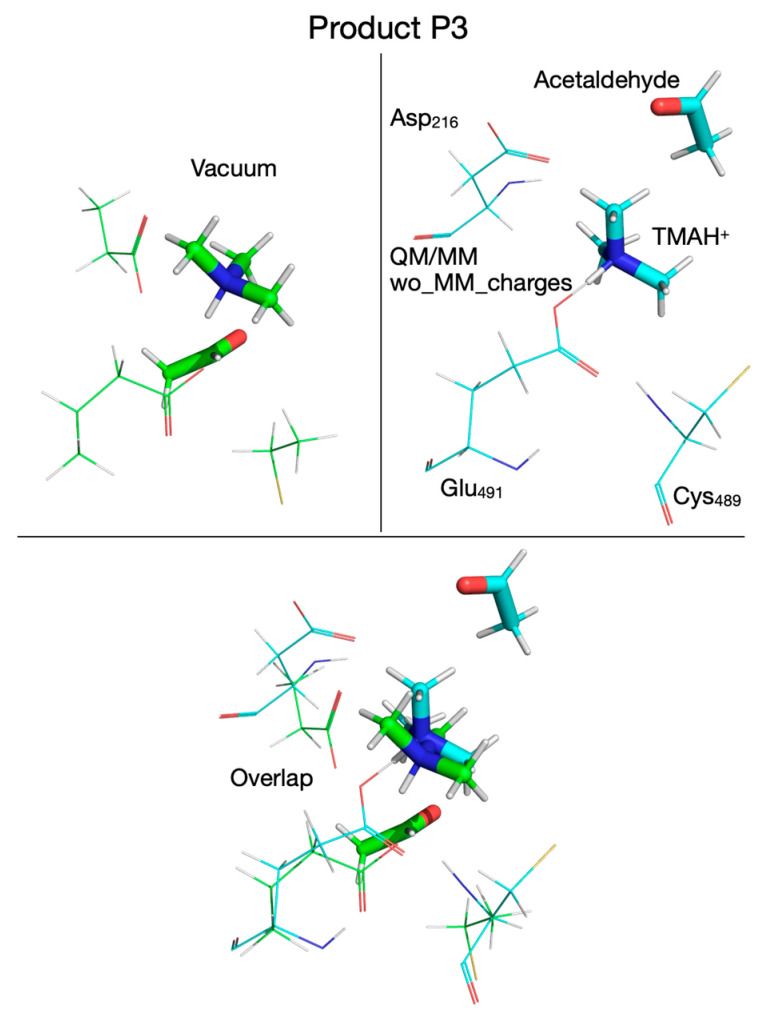
Comparison of product P3 configurations obtained by using the ωB97XD functional and the split-valence double-zeta Def2-SVP basis set in vacuum (**top left**) and in the enzyme environment without embedding the MM partial atomic charges in the QM subsystem (**top right**). The overlap of P3 configurations obtained by the two different calculation setups is shown in the (**bottom**) panel (carbon = green/cyan, oxygen = red, nitrogen = blue, hydrogen = white).

**Figure 21:**
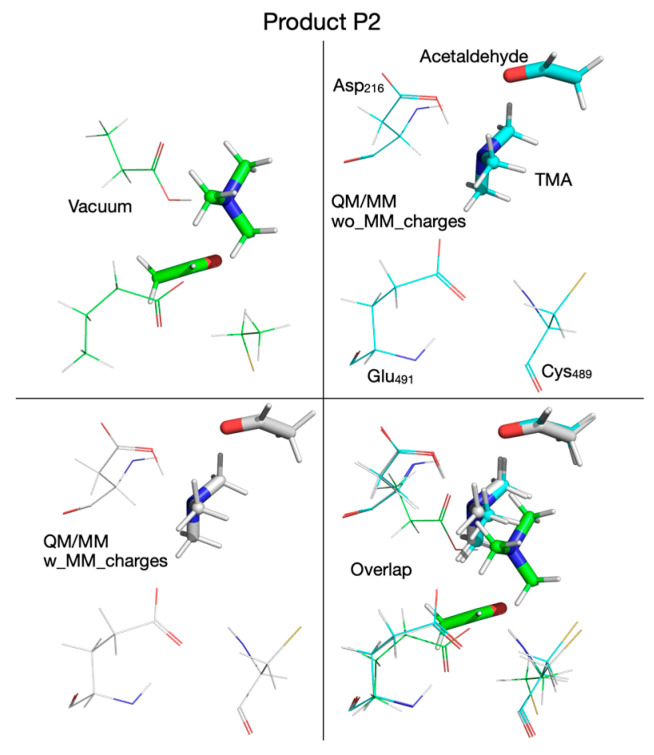
Comparison of product P2 configurations obtained by using the ωB97XD functional and the split-valence double-zeta Def2-SVP basis set in vacuum (**top left**), in the enzyme environment without embedding the MM partial atomic charges in the QM subsystem (**top right**), and by embedding the MM partial atomic charges in the QM subsystem (**bottom left**). The overlap of P2 configurations obtained by the three different calculation setups is shown in the (**bottom-right**) panel (carbon = green/cyan/grey, oxygen = red, nitrogen = blue, hydrogen = white).

**Figure 22:**
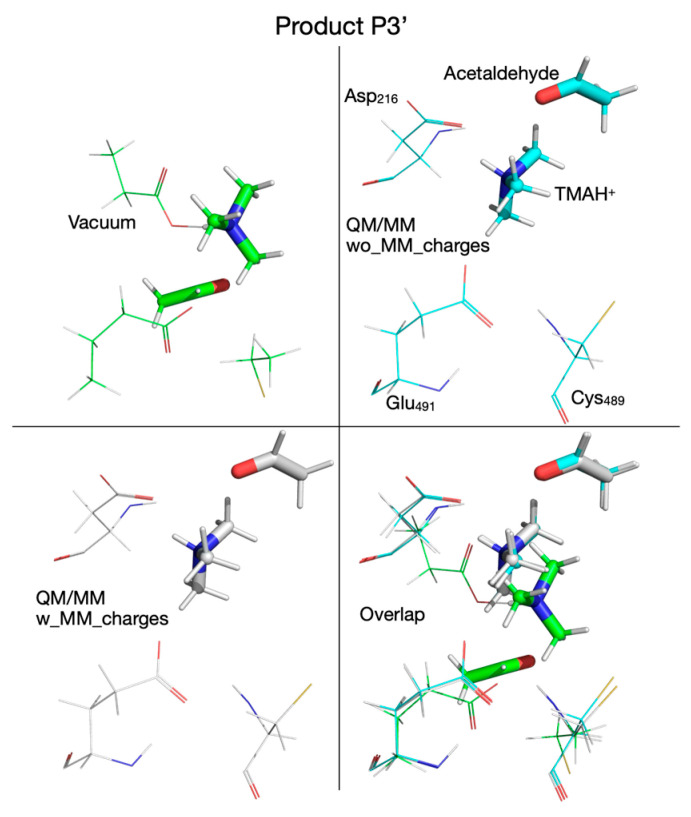
Comparison of product P3’ configurations obtained by using the ωB97XD functional and the split-valence double-zeta Def2-SVP basis set in vacuum (**top left**), in the enzyme environment without embedding the MM partial atomic charges in the QM subsystem (**top right**), and by embedding the MM partial atomic charges in the QM subsystem (**bottom-left**). The overlap of P3’ configurations obtained by the three different calculation setups is shown in the (**bottom-right**) panel (carbon = green/cyan/grey, oxygen = red, nitrogen = blue, hydrogen = white).

When comparing the QM vacuum cluster with QM/MM calculations, for each case discussed below we are referring to Figures that show the reaction mechanism and its energetics in vacuum (e.g., Figure 2) and in the enzyme environment without (Figure 6) and with (Figure 7) MM partial atomic charges embedded. We will also make reference to Figure 8, Figure 9, Figure 10, Figure 11, Figure 12, Figure 13, Figure 14, Figure 15, Figure 16, Figure 17, Figure 18, Figure 19, Figure 20, Figure 21 and Figure 22, which show the calculated structures of the reactant, product, intermediates, and transition states along the reaction path performed in vacuum and with the QM/MM method. Certain features of the reaction mechanism can be traced back to the reaction of the substrate (in this case choline) with the chemical surrogates that mimic the active site residues directly involved in the reaction (we call this a surrogate enzyme environment: Asp_216_ → propionate, Cys_489_ radical → thiol radical, Glu_491_ → butyrate). When comparing the reaction mechanism (vide supra) calculated in vacuum (Figure 2, structures shown in Figure 8, Figure 9, Figure 10, Figure 11, Figure 12, Figure 13, Figure 14, Figure 15, Figure 16, Figure 17, Figure 18, Figure 19, Figure 20, Figure 21 and Figure 22, geometrical parameters and energetics given in Table 1 and Table 2, Supplementary Table S2) with the one calculated by the QM/MM method (Figure 6, Figure 7, Figure 8, Figure 9, Figure 10, Figure 11, Figure 12, Figure 13, Figure 14, Figure 15, Figure 16, Figure 17, Figure 18, Figure 19, Figure 20, Figure 21 and Figure 22, Table 1 and Table 2, Supplementary Table S2) we notice that many reaction steps are identical; however, there are some subtle differences that point to either a steric and/or an electronic effect due to the enzyme (and solvent) environment being present. For example, in the first step of the reaction calculated in vacuum (Figure 2, Figure 8, Figure 9 and Figure 10, top-left panel, Table 1) or with the QM/MM method without embedding the MM partial atomic charges (Figure 6, Figure 8, Figure 9 and Figure 10, top-right panel, Table 1), there is a concerted transfer of a H atom from choline to the Cys_489_ radical and of the hydroxylic proton of choline to Glu_491_, whereas when the calculation is performed with the QM/MM method by embedding the MM partial atomic charges (Figure 7, Figure 8, Figure 9 and Figure 10, bottom-left panel, Table 1), the choline hydroxylic proton is not transferred to Glu_491_ in the first reaction step, pointing to an electronic effect of the enzyme environment on this reaction step. However, the activation energies for the first step in the reaction are closer to each other when the calculation is performed in vacuum (8.5 kcal/mol) or with the QM/MM method by embedding the MM partial atomic charges (9.5 kcal/mol) than when performed without embedding the MM partial atomic charges (6.2 kcal/mol).

The fate of the hydroxylic proton of choline in the first reaction step determines the chemical identity of intermediate 1 (I_1_, Figure 10). When the calculations are performed in vacuum or with the QM/MM method without embedding the MM partial atomic charges, intermediate I_1_ is a radical containing a carbonyl group (Figure 10, top-left and top-right panels, Table 1), while when the MM partial atomic charges are embedded in the calculation, I_1_ is a radical containing an alcohol group (Figure 10, bottom-left panel, Table 1); however, all three I_1_ intermediates are within 1 kcal/mol from each other and ~3 kcal/mol above the reactant (R) (Figure 2 and Figure 6, and 7, Table 2). Transition state 2 (TS_2_) corresponding to the break of the C_2_-N bond in choline, contains TMA and an enol radical (Figure 11, bottom-left panel, Table 1), when calculated with the QM/MM method by embedding the MM partial atomic charges, or TMA and an acetaldehyde radical when calculations are performed in vacuum (Figure 11, top-left panel, Table 1), or with the QM/MM method without embedding the MM partial atomic charges (Figure 11, top-right panel, Table 1). The activation energies for the second step in the reaction are again closer to each other when the calculation is performed in vacuum (9.7 kcal/mol) or with the QM/MM method by embedding the MM partial atomic charges (9.5 kcal/mol) than when performed without embedding the MM partial atomic charges (2.3 kcal/mol). Intermediate 2 (I_2_) is 6.8 and 5.2 kcal/mol above R when calculated in vacuum (Figure 2 and Figure 12, top-left panel, Table 2) and with the QM/MM method without embedding the MM partial atomic charges (Figure 6 and Figure 12, top-right panel, Table 2), respectively, and 11.9 kcal/mol when the MM partial atomic charges are embedded (Figure 7 and Figure 12, bottom-left panel, Table 2).

We note that from this point forward the reaction mechanism calculated with the QM/MM method by embedding the MM partial atomic charges diverges from the one calculated in vacuum or without embedding the MM partial atomic charges. On the PES calculated with the MM partial atomic charges embedded, in the third reaction step, the hydroxylic proton is transferred from the enol radical (I_2_) to Glu_491_ before reaching transition state 2’ (TS_2_’) (Figure 7 and Figure 13, top panel, Supplementary Figure S7, top-right panel) and is transferred back to form the carbinolamine radical (I_2_’) (in which the quaternary nitrogen atom is now bonded to C_1_ atom) after TMA migration (Figure 7 and Figure 13, bottom panel, Table 1). TS_2_’ and I_2_’ have no correspondents on the PES calculated in vacuum or using the QM/MM method without embedding the MM partial atomic charges, pointing to a pure electronic effect of the enzyme environment at this stage of the reaction. The activation energy of TS_2_’ is only 4.0 kcal/mol, and intermediate I_2_’ is 8.3 kcal/mol above R (Figure 7, Table 2). In the third step of the reaction (fourth step when the QM/MM method with embedding the MM partial atomic charges is used), a H atom is transferred back from Cys_489_ to the acetaldehyde radical when calculations are performed in vacuum (Figure 2, Figure 14 top-left panel, Table 1) or with QM/MM without embedding the MM partial atomic charges (Figure 6, Figure 14 top-right panel, Table 1), or to the carbinolamine radical when QM/MM with MM partial atomic charges embedded is used (Figure 7, Figure 14 bottom-left panel, Table 1). The activation energies of transition state 3 (TS_3_) cluster together (13.8, 11.0, 9.9 kcal/mol, when calculations are performed in vacuum or with QM/MM, Table 2), while the relative energies of intermediate 3 (I_3_) with respect to R are negative (−9.2, −5.6, −4.6 kcal/mol, Table 2) when calculations are performed in vacuum (Figure 2, Figure 15 top-left panel, Table 1), with QM/MM without embedding the MM partial atomic charges (Figure 6, Figure 15 top-right panel, Table 1), or with QM/MM with the MM partial atomic charges embedded (Figure 7, Figure 15 bottom-left panel, Table 1), respectively, suggesting that carbinolamine is more stable than choline itself in this environment.

In step four of the reaction (fifth step is when QM/MM with the MM partial atomic charges embedded is used), the C_1_-N bond is broken concomitantly with the transfer of the hydroxylic proton from carbinolamine to Glu_491_ in order to produce the final products (TMA and acetaldehyde). The activation energy for transition state 4 (TS_4_) is 17.2, 10.1, and 24.7 kcal/mol (Table 2), and intermediate 4 (I_4_) is 5.9, 4.1, and 19.6 kcal/mol above R (Table 2) when the calculations are performed in vacuum (Figure 2, Figure 16, Figure 17 top-left panel, Table 1), using QM/MM without embedding the MM partial atomic charges (Figure 6, Figure 16, Figure 17 top-right panel, Table 1), or QM/MM with the MM partial atomic charges embedded (Figure 7, Figure 16, Figure 17 bottom-left panel, Table 1), respectively. We notice that this last step of the reaction is the rate-limiting step when calculations are performed in vacuum or with QM/MM with the MM partial atomic charges embedded, and even when the calculations are performed with QM/MM without embedding the MM partial atomic charges, the activation energy for this step is the second highest (Figure 6). The effects of the enzyme environment on this reaction step are opposite, i.e., the steric (cage) effect on the energetics of both TS_4_ and I_4_ is favorable due to lowering the transition state and intermediate I_4_ on the PES when MM partial atomic charges are not included in calculations, while the electronic effect (i.e., the effect of the electric field of MM partial atomic charges when included) is unfavorable due to significantly elevating TS_4_ and I_4_ above R on the PES.

If TMA and acetaldehyde are the final products of the enzymatic reaction, then the choline hydroxylic proton, which was transferred to Glu_491_ (or perhaps to Asp_216_ possibly via Thr_502_) at some point during the reaction, must be reclaimed by TMA (leading to protonated TMA) for the reaction cycle to be complete and the enzyme to return to its active state ready to start a new reaction cycle. Therefore, we assumed two scenarios for this last step of the enzymatic reaction in which the proton is transferred to TMA from Glu_491_ (or from Asp_216_). To perform the calculations for the first scenario (proton transfer from Glu_491_ to TMA), we rotated TMA in I_4_ such that the lone electron pair of TMA’s N atom points towards Glu_491_ and searched for a transition state for proton transfer from Glu_491_ to TMA in vacuum and in the enzyme environment. The chemical structures involved in this reaction step are shown in Figure 18, Figure 19 and Figure 20. The starting structure for this step (P) is shown in Figure 18 in the top-left panel for the vacuum calculation and the QM/MM without MM partial atomic charges embedded is shown in the top-right panel. The overlap of the two structures is shown in the bottom panel. We could not obtain a structure for P when the MM partial atomic charges were included in calculations because the geometry optimization failed with a “microiteration” error (see Methods). The P configuration is 8 kcal/mol below the reactant (R) when the calculation is performed in vacuum (Figure 4, Table 2) and 8.6 kcal/mol above R when the calculation is performed in the enzyme environment. This is a striking result which indicates that at least the steric (cage) constrains imposed by the enzyme on the active site flips the energetics of this reaction from exothermic to endothermic.

A transition state for proton transfer from Glu_491_ to TMA (TS_5_, Figure 19) was found with the search algorithm only when calculations were performed in vacuum, and this point on the PES was confirmed by frequency calculation to have a single imaginary frequency (77i cm^−1^). The transition state corresponds to the proton being 1.172 Å far from TMA and 1.343 Å away from Glu_491_ (Table 1). When the calculations were performed using the QM/MM without embedding the MM partial atomic charges, the PES decreased monotonically from P to P3 where the proton is bound to TMA (Supplementary Figure S9, top panel, Table 1); therefore, the activation energy for this step is zero at this level of QM calculation (Table 2). The P3 configuration was successfully calculated both in vacuum (Figure 20, top-left panel, Table 1) and with the QM/MM without embedding the MM partial atomic charges (Figure 20, top-right panel, Table 1), and their overlap is shown at the bottom of Figure 20. P3 is 1 kcal/mol below P, which makes this reaction step slightly exothermic, but the overall reaction remains endothermic when the enzyme environment is present (Table 2).

Alternatively, in the second calculation scenario, the hydroxylic proton may be first transferred from Glu_491_ to Asp_216_ via another amino acid (e.g., Thr_502_), and then to TMA. We labeled the configuration in which the proton is bound to Asp_216_ P2 (Figure 21). We were able to calculate this configuration both in vacuum (Figure 21, top-left panel, Table 1) and with QM/MM (Figure 21, top-right panel, bottom-left panel, Table 1), and their overlap is shown in Figure 21 in the bottom-right panel. In vacuum, P2 is 13.5 kcal/mol below R; however, it is above R 7.3 and 13.7 kcal/mol, respectively, when QM/MM without/with embedding the MM partial atomic charges is used (Table 2). We could not find a transition state (TS_6_) for transferring the proton from Asp_216_ to TMA neither in vacuum nor in the enzyme environment. When calculations are performed in vacuum or with the QM/MM without embedding the MM partial atomic charges, the PES climbs from P2 to P3’ monotonically (Supplementary Figures S10 and S11, top panel, Table 1), which makes the activation energy equal to the enthalpy of this step (2.7 and 3.8 kcal/mol, respectively, Table 2). However, when using the QM/MM with the MM partial atomic charges embedded, the PES descends monotonically from P2 to P3’ (Supplementary Figure S12, top panel), which makes the activation energy equal to zero (Table 2). This reaction step is another example in which the steric and electronic effect of the enzyme environment are opposite; the steric effect is unfavorable for the proton being transferred from Asp_216_ to TMA while the enzyme electric field favors the proton transfer.

## 4. Discussion

In this study, we used several quantum mechanical methodologies (ab initio Hartree–Fock and density functional theory) to map out the reaction mechanism of choline-TMA lyase transformation catalyzed by the gut microbial enzyme of *D. alaskensis* CutC. We first investigated the dependence of the reaction mechanism on the size of the QM subsystem using the HF/6-31G(d) ab initio method for three QM systems with 52, 141, and 202 atoms and found that the reaction mechanism is not sensitive to the number of atoms included in the QM system, leading to the same reaction mechanism with the same number of elementary step reactions, same intermediates, and same transition states. In all three QM systems, the rate-limiting step is the same, i.e., the transfer of a H atom from choline to the Cys489 radical, and the activation energy is practically the same (29.2 kcal/mol). Next, we selected the DFT functional ωB97XD and the split-valence double-zeta basis set Def2-SVP to explore the PES of the reaction by performing cluster calculations on a QM system formed from the substrate (choline) and three amino acids (Asp_216_, the Cys_489_ radical, and Glu_491_) that are directly involved in substrate cleavage by participating or assisting in bond breaking/forming, leading to the product of the enzymatic reaction (TMA and acetaldehyde when HF method is used and most likely carbinolamine when the DFT method is used).

With the DFT method, we used three computational protocols which allowed us to dissect and shed light on the effect of enzyme and solvent environments on the reaction mechanism. In the first protocol, we replaced the three amino acids of the QM system with chemical surrogates representing their side chains (Cys_489_ radical → ethylthiol radical, Glu_491_ → butyrate, and Asp_216_ → propionate), restrained the C_α_ atoms at the positions they have in the crystal structure (PDB id: 5FAU), and explored the PES for the reaction through QM cluster calculations in vacuum (Figure 2). This computational scenario produced a reaction mechanism with four intermediates and five transition states which gives us a basic understanding of the chemical interactions between the most relevant amino acid residues in the active site and choline, i.e., which bonds are broken and formed and in which order. In this protocol (Figure 2), the first step of the reaction (R → TS_1_ → I_1_) is a concerted transfer of a H atom from choline to the Cys_489_ radical and of the hydroxylic proton of choline to Glu_491_. In the second step (I_1_ → TS_2_ → I_2_), the C_2_-N bond in the choline radical is broken. In the third step (I_2_ → TS_3_ → I_3_), the H atom is transferred back from Cys_489_ to the acetaldehyde radical and the C_1_-N bond is formed, leading to carbinolamine. In the fourth step (I_3_ → TS_4_ → I_4_), the C_1_-N bond is broken, leading to TMA and acetaldehyde, and in the fifth step (P → TS_5_ → P3), the hydroxylic proton is transferred from Glu_491_ back to TMA. The reaction energetics indicates that the overall reaction is exothermic at this level of QM theory; however, the most surprising finding was the formation along the reaction path of an intermediate (I_3_), containing a choline isomer (carbinolamine) that is more stable than choline itself in this enzyme surrogate environment (Figure 2).

For calculations in the actual enzymatic environment, we used the same QM system as for vacuum cluster calculations and an MM system formed from the atoms of the enzyme monomer and crystallization water. The amino acid residues and solvent molecules within 3.5 Å from the QM subsystem were allowed to move freely during geometry optimization and transition state search. The two computational scenarios used for QM/MM calculations consisted in including (or not) the interactions between the MM partial atomic charges and the wave function of the QM subsystem. Being able to “switch off” these electrostatic interactions allowed us to separate the “steric” (restricting or cage) effect from the “electronic” (polarization) effect of the enzyme on the reaction. The steric effect the enzyme exerts on the reaction comes from the geometrical constraints imposed on the substrate by the enzyme (cage effect), which basically eliminates the entropy contribution due to the substrate’s translational/rotational/librational motion to the energetics of the reaction. In addition, the enzyme provides a tailored cavity and strategically placed amino acid residues that activate the reaction and participate in bond breaking/formation elementary steps that define the reaction mechanism. When this computational protocol was used, the reaction mechanism obtained (Figure 6) was almost identical with the one obtained in vacuum (Figure 2), except for step three of the reaction in which carbinolamine is deprotonated, with the hydroxylic proton being bonded to Glu_491_. The energetics of the reaction are somewhat different, with the most puzzling fact being that the reaction is now overall endothermic, pointing to the fact that breaking the C_1_-N bond in carbinolamine might not be favored by the enzyme and carbinolamine may in fact be the final product of the enzymatic reaction [50,51], which probably degrades to protonated TMA and acetaldehyde after its release in water [50].

When the MM partial atomic charges are included in the QM/MM calculations in the third computational protocol, the first reaction step is not a concerted H atom/proton transfer anymore; only the H atom is being transferred from choline to the Cys_489_ radical. Also, the reaction mechanism adds another reaction step in which the configuration of intermediate I_2_ is very similar to transition state TS_2_ (Figure 7). The activation energy to break the C_1_-N bond in carbinolamine is the largest in this reaction, making this step the rate-limiting step. The reaction energetics calculated with this protocol makes the reaction even more endothermic (Figure 7). This suggests that both the steric and electronic effects of the enzyme environment do not favor breaking the C_1_-N bond in carbinolamine. It is worth noting that, in this study, we limited our theoretical investigation of this enzymatic reaction to exploring the potential energy surface only, that is, without any contribution from the kinetic energy of the atoms, in part because we opted to focus on and compare several QM methodologies which produced a wealth of computational results that needed to be presented and discussed in detail. Including protein dynamics through molecular dynamics simulations would have made the presentation even more convoluted and would have lengthened the manuscript considerably. In addition, and probably more relevant to our choice, previously published studies pointed out that protein dynamics contribution to enzymatic catalysis either does not exist or is not well understood [79].

Earlier theoretical work aimed at understanding the reaction mechanism for the choline-TMA lyase using a combined QM/MM approach was performed by Yang et al. [49] in which the QM system contained 281 atoms with a net charge of zero. For the QM region, the level of quantum theory used was the DFT functional ωB97Eh [80] using the split-valence double-zeta basis set 6-31G(d) [81], with electrostatic embedding as implemented in the program TeraChem [82], and the Amber ff14SB force field [83] to describe the region of the protein and solvent not included in the QM system. The authors focused on two reaction steps: the formation of the short-lived choline radical intermediate and its deprotonation. Using steered molecular dynamics simulations (SMD), the authors observed a concerted H atom transfer from choline to the Cys_489_ radical and a proton transfer from choline to Glu_491_. The proton transfer follows an increase in acidity of choline hydroxyl proton after the H atom transfer, which leads to a reduction in the pKa value of the α-hydroxy radical due to the formation of a stable ketyl radical [84]. Yang et al. [49] concluded that the presence of Glu_491_ is required as a base in order for the reaction to advance towards its final products. The authors also observe in their simulations the cleavage of the C-N bond with the formation of TMA and acetaldehyde, concluding that the enzymatic reaction favors the direct elimination mechanism hypothesized by Bodea et al. [36]. However, the authors of this study acknowledged that the simulation method (SMD) implemented does not produce accurate activation energies but only approximate geometrical configurations of the intermediates along the reaction path.

More relevant to the results presented in this manuscript are the more recently published QM/MM studies of the CutC reaction mechanism from Yang et al. [50] and Hanzevacki et al. [51], which both address the following unanswered questions: (1) Does Glu_491_ play the role of a base abstracting a proton from choline? (2) Is the final product of the enzymatic reaction TMA and acetaldehyde? While both groups concluded that the reaction proceeds with TMA migration and the final product of the enzyme is carbinolamine, Yang et al. [50] suggested that Glu_491_ is not required to function as a base because the choline hydroxylic proton is never transferred to Glu_491_ during the reaction and is only helping positioning choline in the active site such that one of the H atoms bonded to its C_1_ atom is close to the S atom of the Cys_489_ radical (needed for the H atom transfer to occur). On the other hand, Hanzevacki et al. [51] concluded that Glu_491_ is acting as a base and that the hydroxylic proton of choline is transferred to Glu_491_ in the first step of the reaction concerted with the H atom transfer from choline to the Cys_489_ radical. These authors [51] reported the activation energy for the rate-limiting step of the enzymatic reaction to be 13.5 kcal/mol, calculated at the CCSD(T) level of ab initio theory, and found their value to agree with the activation free energy (14.6 kcal/mol) estimated from k_cat_ for the reaction (157 ± 2 s^−1^) reported by Bodea et al. [36]. Our QM/MM calculations produced an activation energy for the same reaction step (the transfer of the H atom back from Cys) in the range of 9.9–11.3 kcal/mol depending on the basis set used (Figure 7).

Regarding the computational methodology employed, these two recently published QM/MM studies described the interactions between atoms in the QM region with the DFT functional B3LYP corrected for lack of dispersion interactions through the Grimme’s D3 empirical method with BJ damping [63,64]. As a basis set, they used either Pople’s split-valence double-zeta basis set 6-31G(d) (Hanzevacki et al. [51]) or Ahlrichs’ split-valence double-zeta basis set Def2-SVP (Yang et al. [50]) as implemented in the ORCA program [85]. The MM subsystem of the QM/MM system was described with the Amber ff14SB force field [83] as implemented in the DL_POLY program [86].

While the present studies, along with the work of Yang et al. [50] and Hanzevacki [51], all focus on understanding the reaction mechanism of choline-TMA lyase by CutC enzyme using QM/MM calculations, there are several significant differences among them. For example, both Yang et al. [50] and Hanzevacki et al. [51] mapped the PES using a single DFT method (B3LYP-D3 [59,60,63]) and subsequently performed only single-point calculations using either other DFT functionals (M06-2X [61]) or a more sophisticated ab initio method (coupled-cluster, CCSD(T) [87]), assuming that the choice of computational methodology does not affect the geometries of reactant, intermediates, transition states, and product. In contrast, we mapped the PES using multiple QM methodologies (i.e., the Hartree–Fock ab initio method and three DFT functionals: B3LYP [59,60] and M06-2X [61] with/without dispersion interaction correction [63], and ωB97XD [62]), dissected the enzyme environment into steric (cage) and electronic effects, and used this decomposition to explain differences observed in the reaction mechanisms obtained with various computational protocols. One of the most surprising results of this work is that there is a fundamental difference between the reaction mechanism obtained with an ab initio QM method (HF in this case) and the DFT method. The reaction mechanism obtained with the HF method is consistent with the reaction mechanism proposed by Bodea et al. [36] following experimental work, which concludes that TMA and acetaldehyde are the final products of the enzymatic reaction. On the other hand, all DFT calculations (cluster and enzyme) point to carbinolamine as the final product of the reaction. Our QM/MM studies point to the conclusion that the steric effect favors Glu_491_ acting as a base for most of the reaction pathway (Figure 6), while the electronic effect favors Glu_491_ acting as a base only in reaction steps three (formation of the carbinolamine radical) and 5 (breaking the C_1_-N bond) (Figure 7). We also conclude that neither the steric nor the electronic effect favor TMA and acetaldehyde as final products of the reaction as they make the reaction significantly endothermic and create a high activation energy barrier for breaking the C_1_-N bond in carbinolamine.

Experimentally, TMA and acetaldehyde have been directly observed to be “final” products of the choline-TMA reaction by CutC [2,10,19,36,88]. Therefore, it was suggested by Yang et al. [50] that carbinolamine quickly degrades to TMA and acetaldehyde in water environment, an exothermic reaction with a calculated activation energy of 18 kcal/mol. We note that in our calculations, breaking the C_1_-N bond in carbinolamine in the enzyme environment is endothermic (12.2 kcal/mol), while the activation energy (24.7 kcal/mol) is higher than for the decomposition of carbinolamine in water into TMA and acetaldehyde, which makes this latter reaction plausible and could account for the experimental observation of TMA and acetaldehyde as final products.

## 5. Conclusions

Being able to dissect and shed light on the effect of the enzyme environment on the reaction mechanism of choline-TMA lyase by the gut microbial enzyme CutC is an insightful approach in understanding the reaction mechanism of this enzyme and a significant contribution of this study. Our theoretical results obtained with the DFT method agree with two recently published QM/MM investigations of the reaction mechanism of choline-TMA lyase by CutC that the final product of enzyme catalysis seems to be carbinolamine and not TMA and acetaldehyde. However, we show that carbinolamine can be further degraded to TMA and acetaldehyde by CutC, but the enzyme makes this last step endothermic, requiring a rather high activation energy which makes this last reaction step less probable to occur within the enzyme. The fact that the HF method favors TMA/acetaldehyde as products and not carbinolamine may point to a fundamental difference between the ab initio and DFT methods for this particular enzymatic system and perhaps a need for further theoretical explorations (e.g., using the MP2 approach).

We also agree with the conclusion of Hanzevacki et al. [51] that Glu_491_ plays the role of a base in this reaction by temporarily abstracting a proton from the hydroxylic group of choline sometime during the reaction, and this proton transfer seems to be critical for the reaction to proceed to completion. We also note that the choice of computational protocol alters the reaction energetics to some extent but can also affect the identity and precise geometrical configuration of the chemical species appearing along the reaction path.

## Figures and Tables

**Table 1:** Relevant bond distances within reactant, product, reaction intermediates, and transition states along the reaction pathway calculated in vacuum and with the QM/MM method using the DFT functional ωB97XD and the split-valence double-zeta basis set Def2-SVP.

Pair of Atoms	Distance [Å]		
	Vacuum	QM/MM	
		Without MM Charges	with MM Charges
Reactant			
Chol-O…H	1.012	1.037	1.006
Chol-OH…OE-Glu_491_	1.573	1.473	1.584
Chol-C_1_…N	2.582	2.560	2.555
Chol-C_2_…N	1.506	1.507	1.508
Chol-C_1_…H_11_	1.104	1.116	1.115
Chol-C_2_…H_11_	2.174	2.096	2.102
Chol-H_11_…S-Cys_489_	3.780	2.468	2.453
Transition state 1 (TS_1_)			
Chol-O…H	1.396	1.420	1.055
Chol-OH…OE-Glu_491_	1.064	1.053	1.419
Chol-C_1_…N	2.559	2.560	2.538
Chol-C_2_…N	1.514	1.525	1.524
Chol-C_1_…H_11_	1.393	1.369	1.459
Chol-C_2_…H_11_	2.161	2.214	2.312
Chol-H_11_…S-Cys_489_	1.597	1.643	1.559
Intermediate 1 (I_1_)			
Chol-O…H	1.419	1.421	1.049
Chol-OH…OE-Glu_491_	1.054	1.059	1.436
Chol-C_1_…N	2.581	2.590	2.535
Chol-C_2_…N	1.573	1.599	1.542
Chol-C_1_…H_11_	2.685	2.336	2.283
Chol-C_2_…H_11_	3.232	2.904	2.941
Chol-H_11_…S-Cys_489_	1.366	1.360	1.360
Transition state 2 (TS_2_)			
Chol-O…H	1.534	1.503	1.053
Chol-OH…OE-Glu_491_	1.020	1.032	1.413
Chol-C_1_…N	2.172	2.748	2.824
Chol-C_2_…N	2.720	1.876	2.751
Chol-C_1_…H_11_	2.859	2.658	2.560
Chol-C_2_…H_11_	3.056	2.956	2.281
Chol-H_11_…S-Cys_489_	1.355	1.350	1.354
Intermediate 2 (I_2_)			
Chol-O…H	1.527	1.546	1.047
Chol-OH…OE-Glu_491_	1.026	1.016	1.425
Chol-C_1_…N	2.958	2.906	2.876
Chol-C_2_…N	2.116	2.268	2.819
Chol-C_1_…H_11_	2.780	2.725	2.535
Chol-C_2_…H_11_	3.020	2.741	2.263
Chol-H_11_…S-Cys_489_	1.349	1.350	1.354
Transition state 2’ (TS_2_’)			
Chol-O…H	-	-	1.347
Chol-OH…OE-Glu_491_	-	-	1.085
Chol-C_1_…N	-	-	2.101
Chol-C_2_…N	-	-	2.700
Chol-C_1_…H_11_	-	-	2.709
Chol-C_2_…H_11_	-	-	2.158
Chol-H_11_…S-Cys_489_	-	-	1.354
Intermediate 2’ (I_2_’)			
Chol-O…H	-	-	1.031
Chol-OH…OE-Glu_491_	-	-	1.490
Chol-C_1_…N	-	-	1.597
Chol-C_2_…N	-	-	2.523
Chol-C_1_…H_11_	-	-	2.822
Chol-C_2_…H_11_	-	-	2.205
Chol-H_11_…S-Cys_489_	-	--	1.353
Transition state 3 (TS_3_)			
Chol-O…H	1.605	1.570	1.042
Chol-OH…OE-Glu_491_	1.008	1.004	1.445
Chol-C_1_…N	2.485	2.432	1.600
Chol-C_2_…N	2.724	2.887	2.555
Chol-C_1_…H_11_	2.292	2.300	2.418
Chol-C_2_…H_11_	1.462	1.490	1.549
Chol-H_11_…S-Cys_489_	1.516	1.495	1.474
Intermediate 3 (I_3_)			
Chol-O…H	1.022	1.418	1.018
Chol-OH…OE-Glu_491_	3.047	1.057	1.540
Chol-C_1_…N	1.568	1.731	1.576
Chol-C_2_…N	2.567	2.600	2.544
Chol-C_1_…H_11_	2.109	2.130	2.116
Chol-C_2_…H_11_	1.099	1.099	1.097
Chol-H_11_…S-Cys_489_	4.720	2.511	2.565
Transition state 4 (TS_4_)			
Chol-O…H	1.736	1.652	1.554
Chol-OH…OE-Glu_491_	0.989	0.995	1.012
Chol-C_1_…N	3.141	3.130	2.901
Chol-C_2_…N	3.224	3.234	3.329
Chol-C_1_…H_11_	2.140	2.115	2.103
Chol-C_2_…H_11_	1.100	1.099	1.097
Chol-H_11_…S-Cys_489_	5.677	3.294	4.343
Intermediate 4 (I_4_)			
Chol-O…H	1.175	1.649	1.558
Chol-OH…OE-Glu_491_	0.990	0.995	1.014
Chol-C_1_…N	3.698	3.150	2.997
Chol-C_2_…N	3.120	3.211	3.487
Chol-C_1_…H_11_	2.135	3.126	2.158
Chol-C_2_…H_11_	1.100	1.098	1.097
Chol-H_11_…S-Cys_489_	5.463	3.894	2.643
Product P			
Chol-OH…OE-Glu_491_	1.017	1.029	-
Chol-OH…OD-Asp_216_	3.255	4.735	-
Chol-OH…N	1.673	1.696	-
Transition state 5 (TS_5_)			
Chol-OH…OE-Glu_491_	1.343	-	-
Chol-OH…OD-Asp_216_	2.976	-	-
Chol-OH…N	1.172	-	-
Product P3			
Chol-OH…OE-Glu_491_	1.542	1.444	-
Chol-OH…OD-Asp_216_	2.665	4.215	-
Chol-OH…N	1.089	1.129	-
Product P2			
Chol-OH…OE-Glu_491_	3.500	4.660	4.702
Chol-OH…OD-Asp_216_	1.050	1.011	1.011
Chol-OH…N	1.556	1.771	1.603
Product P3’			
Chol-OH…OE-Glu_491_	3.278	4.034	4.032
Chol-OH…OD-Asp_216_	1.546	1.535	1.637
Chol-OH…N	1.051	1.069	1.060

The atom distances listed in the table are for reactant (R), products (P, P2, P3, P3’), intermediates (I_1_, I_2_, I_2_’, I_3_, I_4_), and transition states (TS_1_, TS_2_, TS_2_’, TS_3_, TS_4_, TS_5_) found on the reaction pathway of choline-TMA lyase. The structures were obtained through QM calculations (ωB97XD/Def2-SVP level of QM theory) in vacuum and QM/MM calculations (ωB97XD/Def2-SVP level of QM theory and the Amber MM force field) in the enzyme environment with or without embedding the MM partial atomic charges in the calculation of the QM subsystem (See Methods).

**Table 2:** Relative energies and the lowest vibrational frequency for reactant, product, reaction intermediates, and transition states along the reaction pathway calculated in vacuum and with the QM/MM method using the DFT functional ωB97XD and the split-valence double-zeta basis set Def2-SVP.

Chemical Species	Vacuum		QM/MM			
			Without MM Charges		with MM Charges	
	Energy ^a^	Vib Freq ^b^	Energy	Vib Freq	Energy	Vib Freq
Reactant	0	11.9	0	30.7	0	28.5
TS_1_	8.5	737.7i	6.2	773.5i	9.6	1073.9i
I_1_	2.7	11.4	3.5	32.5	2.6	22.8
TS_2_	12.4	130.7i	5.8	-	12.1	-
I_2_	6.8	9.0	5.2	32.8	11.9	34.2
TS_2_’	-	-	-	-	15.9	152.3i
I_2_’	-	-	-	-	8.3	32.0
TS_3_	20.7	1197.8i	16.2	1265.8i	18.2	1039.6i
I_3_	−9.2	10.6	−5.6	33.6	−4.6	34.8
TS_4_	8.1	97.8i	4.5	-	20.0	24.5i
I_4_	5.9	8.1	4.1	31.0	19.6	33.1
P	−8.9	6.2	8.6	30.5	11.8	-
TS_5_	−5.2	77.1i	8.6	-	11.80	-
P3	−5.3	9.7	7.6	30.2	3.7	-
P2	−13.5	9.5	7.3	28.4	13.7	-
P3’	−10.8	-	11.1	-	5.1	-

^a^ Relative energy with respect to reactant in kcal/mol. ^b^ Lowest vibrational frequency in cm^−1^. An imaginary vibrational frequency is a complex number. The relative energies and vibrational frequencies listed in the table are for reactant (R), products (P, P2, P3, P3’), intermediates (I_1_, I_2_, I_2_’, I_3_, I_4_), and transition states (TS_1_, TS_2_, TS_2_’, TS_3_, TS_4_, TS_5_) found on the reaction pathway of choline-TMA lyase. The structures were obtained through QM calculations (ωB97XD/Def2-SVP level of QM theory) in vacuum and QM/MM calculations (ωB97XD/Def2-SVP level of QM theory and the Amber MM force field) in the enzyme environment with or without embedding the MM partial atomic charges in the calculation of the QM subsystem (See Methods).

## Data Availability

All data produced in this research were included in the main manuscript and the Supplementary Information. No additional data is available; however, the reader should contact the corresponding author if clarifications regarding the material presented in this manuscript are needed.

## Supplementary Materials

The following supporting information can be downloaded at: https://www.mdpi.com/article/10.3390/biom16010037/s1, Figure S1: Reaction mechanism and potential energy surface for breaking choline into TMA and acetaldehyde by CutC enzyme obtained from calculations performed in vacuum at Hartree–Fock level of ab initio theory using the 6-31G(d) basis set; Figure S2: The active site of *D. alaskensis* CutC enzyme (PDB id: 5FAU).; Figure S3: Scheme of the reaction mechanism from reactant (choline) to products (TMA and acetaldehyde); Figure S4: Search for transition state for the second reaction step, breaking the C_2_-N bond in choline radical (TS_2_); Figure S5: Search for transition state for the third reaction step, breaking the C_1_-N bond in carbinolamine (TS_4_); Figure S6: Search for the transition state of the second reaction step, breaking the C_2_-N bond in the choline radical (TS_2_); Figure S7: Search for the transition state of the third reaction step, the formation of carbinolamine radical (TS_2_′); Figure S8: Search for transition state for the fifth reaction step, breaking the C_1_-N bond in carbinolamine (TS_4_) to produce TMA and acetaldehyde; Figure S9: Search for the transition state for the proton transfer from Glu_491_ to TMA (TS_5_); Figure S10: Search for the transition state for proton transfer from Asp_216_ to TMA (TS_6_); Figure S11: Search for the transition state for proton transfer from Aspu_216_ to TMA (TS_6_); Figure S12: Search for the transition state for proton transfer from Aspu_216_ to TMA (TS_6_); Table S1: Relevant bond distances within intermediate 2 and transition state 3 that discriminate between these structures when calculated in vacuum with various DFT functionals and the split-valence double-zeta basis set Def2-SVP; Table S2: Electronic energies for reactant, product, reaction intermediates, and transition states along the reaction pathway calculated in vacuum and with the QM/MM method using the DFT functional ωB97XD and the split-valence double-zeta basis set Def2-SVP.
