# Supplementary material for: Steric and Electronic Effects in the Enzymatic Catalysis of Choline-TMA Lyase
Source: Biomolecules. 2025 Dec 25;16(1):37. doi: 10.3390/biom16010037 (PMC12838924; doi:10.3390/biom16010037)
Supplement: Supplementary file 1 [file biomolecules-16-00037-s001.zip › biomolecules-4011044-supplementary.pdf]

Supplementary material

Steric and electronic effects in the enzymatic catalysis of choline-TMA lyase

Valentin Gogonea[1,2,3] and Stanley L. Hazen[1,2,3,4,5]

[1] Department of Heart, Blood & Kidney Research, Cleveland Clinic, Cleveland, 44195, OH, USA

[2] Department of Chemistry, Cleveland State University, Cleveland, 44195, OH, USA

[3] Center for Microbiome and Human Health, Cleveland Clinic, Cleveland, 44195, OH, USA

[4] Department of Cardiovascular Medicine, Heart, Vascular and Thoracic Institute, Cleveland Clinic, Cleveland, 44195, OH, USA

[5] Department of Molecular Medicine, Cleveland Clinic Lerner College of Medicine of Case Western Reserve University School of Medicine, Cleveland, 44195, OH, USA

CORRESPONDING AUTHOR:

Valentin Gogonea, PhD, Department of Chemistry, Cleveland State University, 2121 Euclid Ave., Cleveland, 44195, OH, USA. Tel.: 216-225-2195; Fax: 216-687-9298; Email: v.gogonea@csuohio.edu

Supplementary Figures

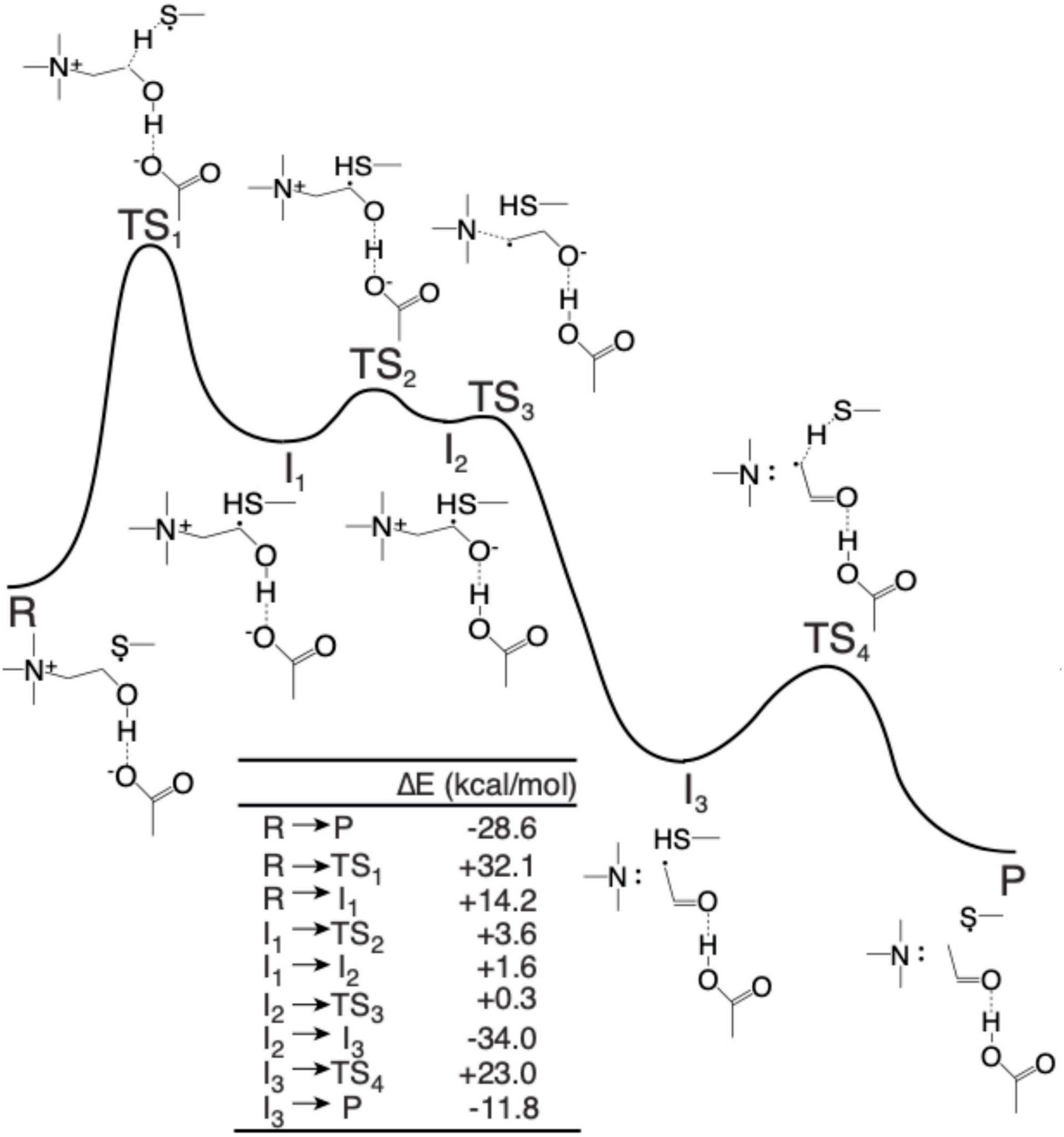

	ΔE (kcal/mol)
R → P	-28.6
R → TS_1	+32.1
R → I_1	+14.2
I_1 → TS_2	+3.6
I_1 → I_2	+1.6
I_2 → TS_3	+0.3
I_2 → I_3	-34.0
I_3 → TS_4	+23.0
I_3 → P	-11.8

Supplementary Figure S1. Reaction mechanism and potential energy surface for breaking choline into TMA and acetaldehyde by CutC enzyme obtained from calculations performed in vacuum at Hartree-Fock level of *ab initio* theory using the 6-31G(d) basis set as implemented in the Gamess program. The QM system is composed of choline, thiol radical and acetate (as surrogates for Cys_{489} and Glu_{491}). The table lists activation energies (for transition states) and relative reaction energies for intermediates and the product along the reaction pathway.

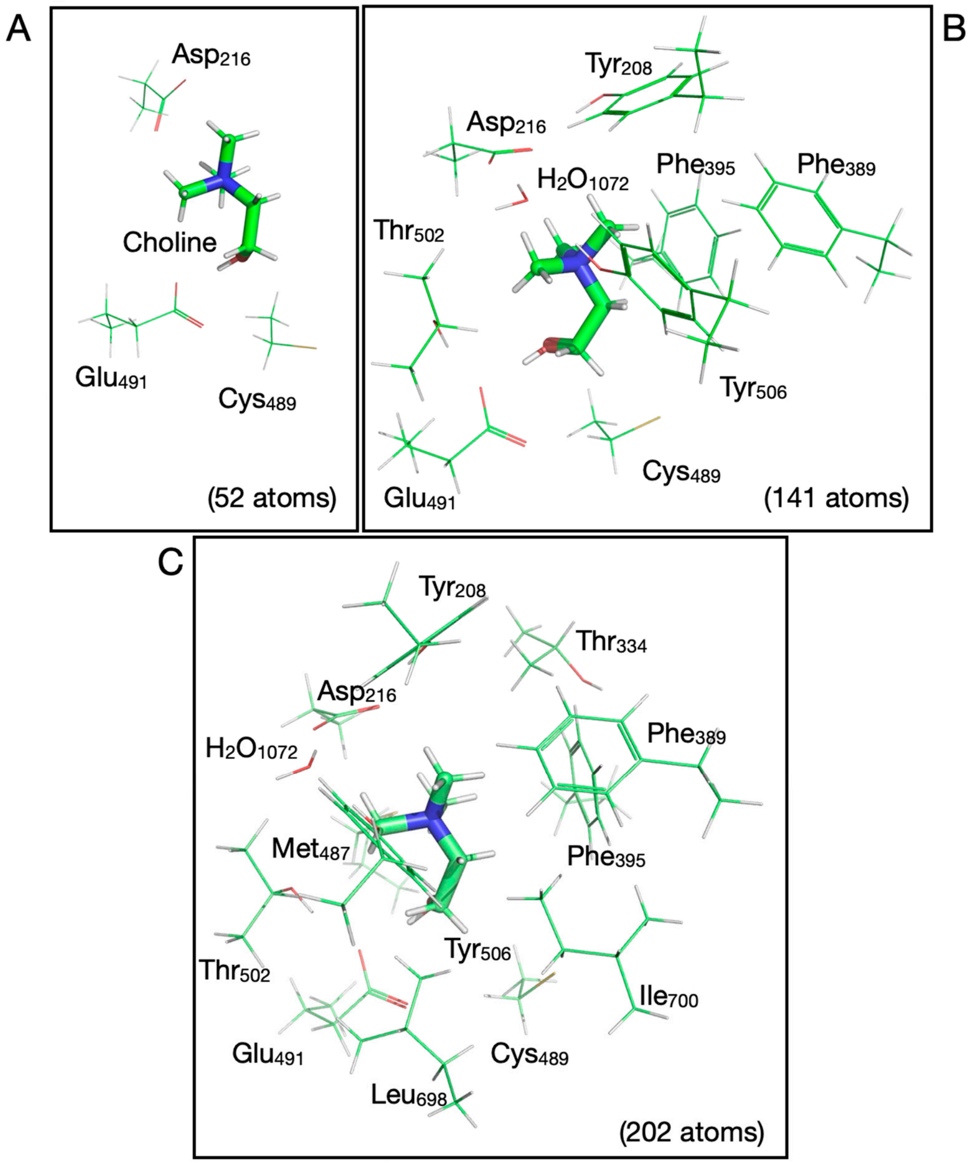

Supplementary Figure S2. The active site of *D. alaskensis* CutC enzyme (PDB id: 5FAU). The amino and carbonyl groups of the terminal residues included in each of the three QM systems shown in this figure were substituted with H atoms and the C_α atoms were frozen during geometry optimizations or transition sate searches to prevent the residues from shifting away from their location in the crystal structure. A. CholineActiveSite-1: CutC active site includes in the QM region three residues (Asp_{216}, Cys_{489}, Glu_{491}) which are involved in bond-breaking and bond formation with choline during the reaction of converting choline into TMA and acetaldehyde. This QM region has 52 atoms. B. CholineActiveSite-2: CutC active site includes in this QM region eight residues and a water molecule (141 atoms). C. CholineActiveSite-3: CutC active site includes in this QM region 12 residues and a water molecule (202 atoms). Choline is drawn with sticks while the active site residues and water are drawn with lines.

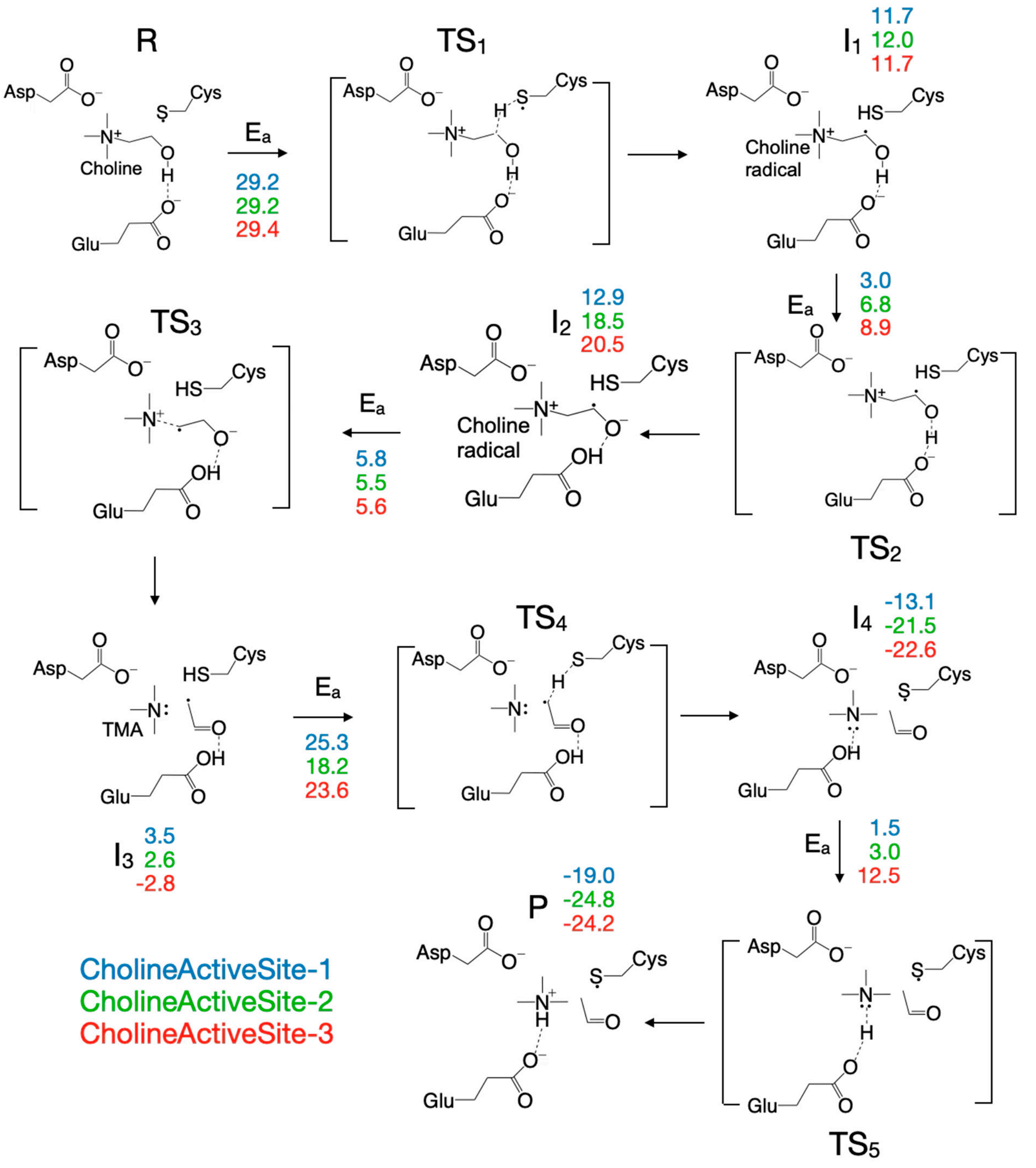

Supplementary Figure S3. Scheme of the reaction mechanism from reactant (choline) to products (TMA and acetaldehyde) with intermediates and transition states for the QM systems shown in Supplementary Figure 2. The potential energy surface was calculated in vacuum at Hartree-Fock level of *ab initio* theory using the 6-31G(d) basis set. Activation energies and reaction energies are listed color-coded as follows: blue font values correspond to calculations performed on CholineActiveSite-1, green font values correspond to calculations performed on CholineActiveSite-2, and red font values correspond to calculations performed on CholineActiveSite-3.

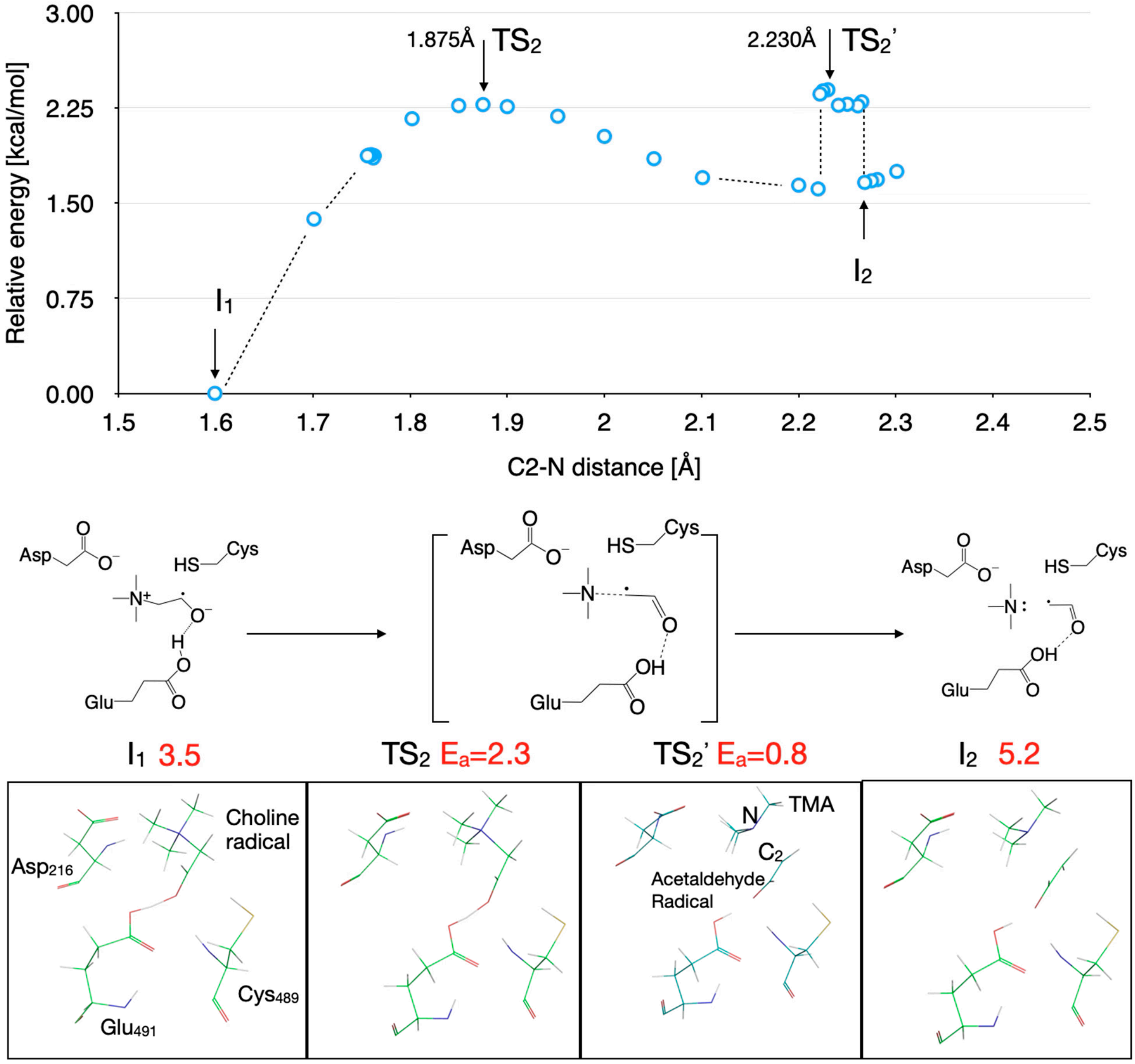

Supplementary Figure S4. Search for transition state for the second reaction step, breaking the C_2-N bond in choline radical (TS_2). The potential energy surface was calculated in enzyme environment with the ωB97XD functional and the split-valence double-zeta basis set Def2-SVP without including the MM partial atomic charges into the QM system. The path for this reaction step was determined by performing geometry optimizations for a series of fixed distances between choline C_2 and N atoms along the reaction coordinate (the distance between C_2 and N). Two transition states were found at C_2-N distances of 1.875 Å (TS_2, 2.3 kcal/mol) and 2.230 Å (TS_2', 0.8 kcal/mol).

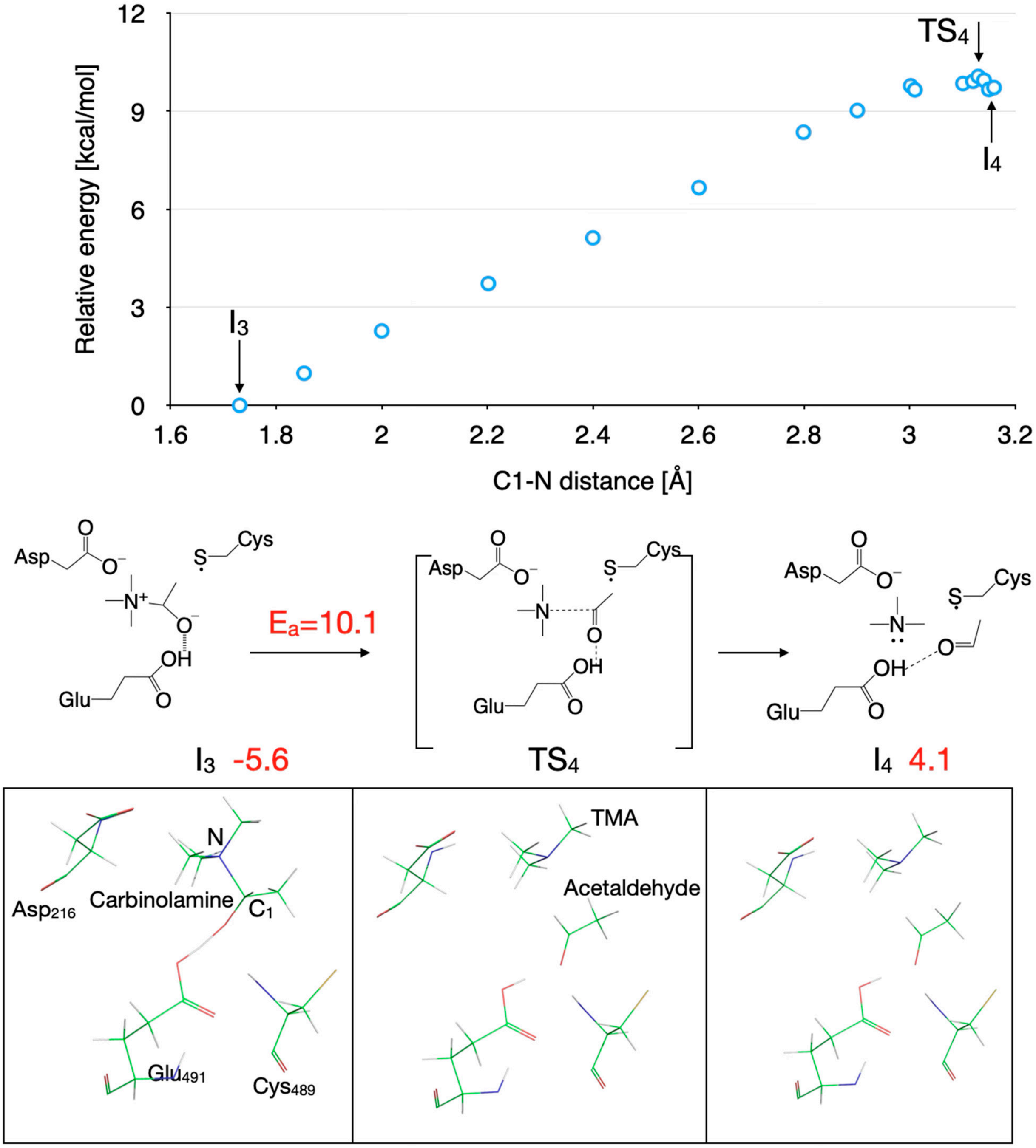

Supplementary Figure S5. Search for transition state for the third reaction step, breaking the C_1-N bond in carbinolamine (TS_4). The potential energy surface was calculated in enzyme environment with the ωB97XD functional and the split-valence double-zeta basis set Def2-SVP without including the MM partial atomic charges into the QM system. The path for this reaction step was determined by performing geometry optimizations for a series of fixed distances between choline C_1 and N atoms along the reaction coordinate (the distance between C_1 and N). The transition state was found at C_1-N distance of 3.130 Å (TS_4, 10.1 kcal/mol).

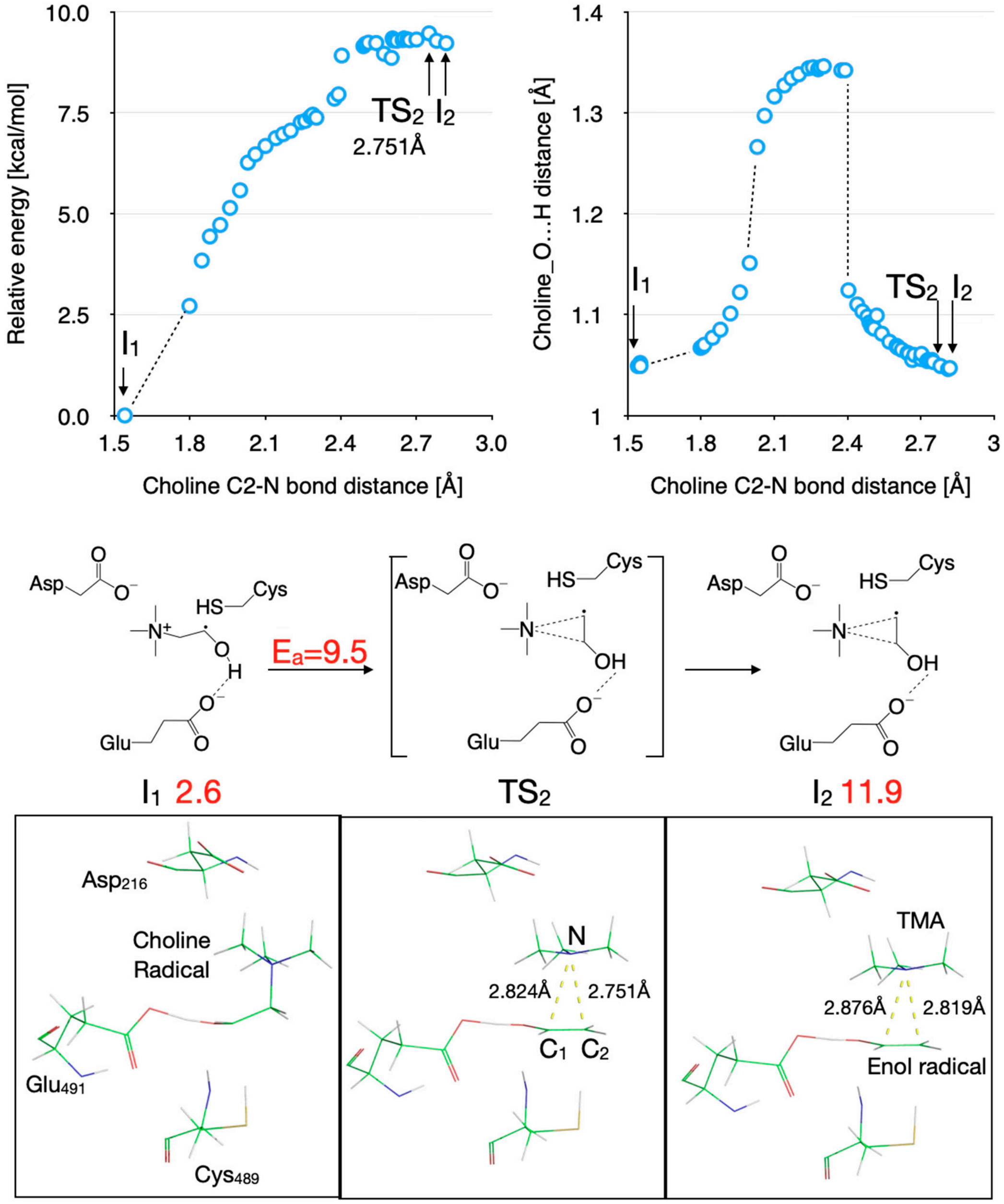

Supplementary Figure S6. Search for the transition state of the second reaction step, breaking the C_2-N bond in the choline radical (TS_2). The potential energy surface was calculated in enzyme environment with the ωB97XD functional and the split-valence double-zeta basis set Def2-SVP by including the MM partial atomic charges into the QM subsystem and performing geometry optimizations for a series of fixed distances between choline C_2 and N atoms along the reaction coordinate (the distance between C_2 and N). The transition state (TS_2) has an activation energy of 9.5 kcal/mol and was found at the C_2-N distance of 2.623 Å. The top-right panel shows the change in the distance between the H and O atoms of the hydroxyl group of the choline radical. The graph shows that the proton is quickly transferred back-and-forth to Glu_{491} before reaching the transition state.

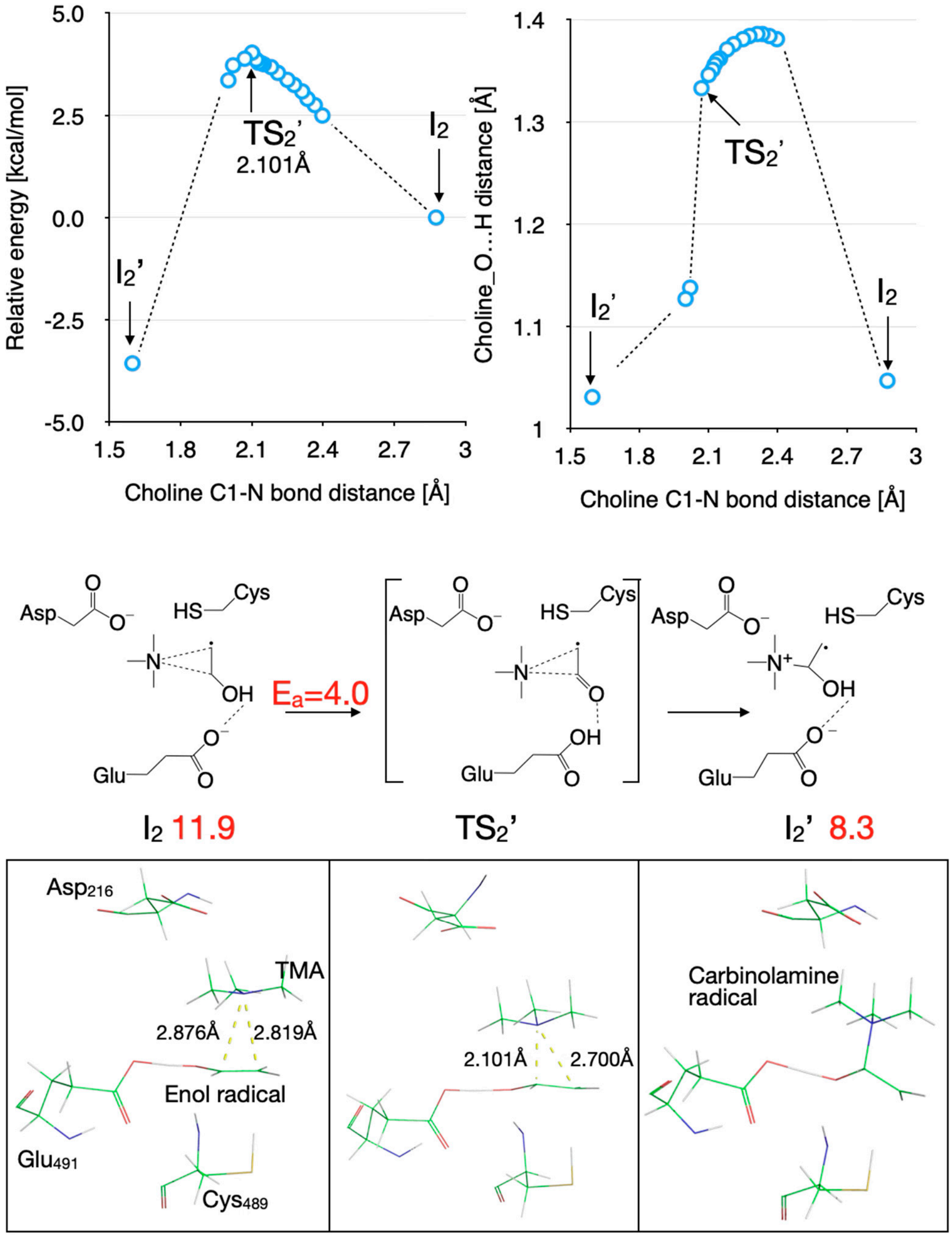

Supplementary Figure S7. Search for the transition state of the third reaction step, the formation of carbinolamine radical (TS_2'). The potential energy surface was calculated in enzyme environment with the ωB97XD functional and the split-valence double-zeta basis set Def2-SVP by including the MM partial atomic charges into the QM subsystem and performing geometry optimizations for a series of fixed distances between choline C_1 and N atoms along the reaction coordinate (the distance between C_1 and N). The transition state TS_2' was found at C_1-N distance of 2.101 Å and has an activation energy of 4.0 kcal/mol. The top-right graph shows that the proton from the hydroxyl group of the enol radical is transferred to Glu_{491} before reaching the transition state but then is transferred back to the O atom of the acetaldehyde radical leading to intermediate I_2'.

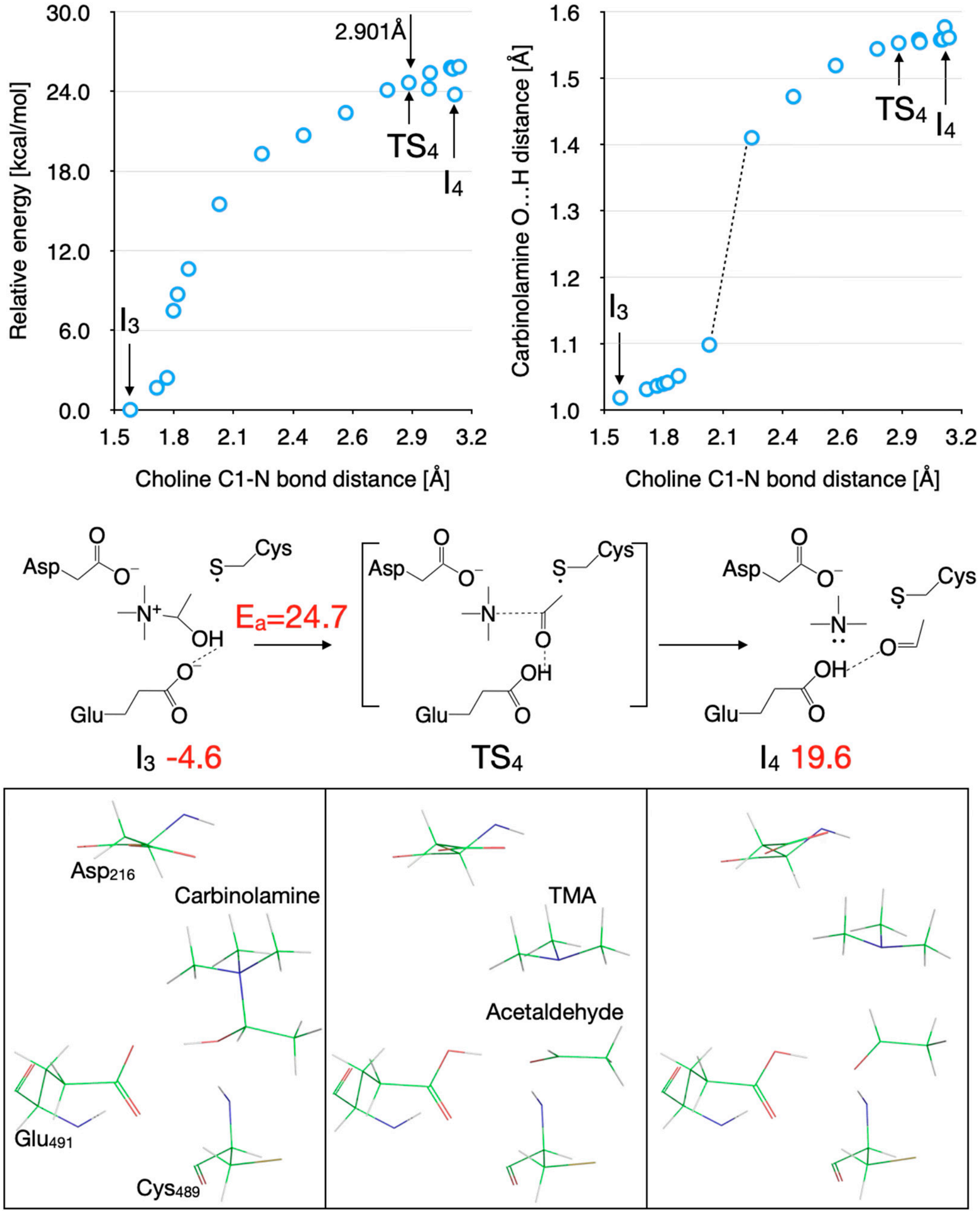

Supplementary Figure S8. Search for transition state for the fifth reaction step, breaking the C_1-N bond in carbinolamine (TS_4) to produce TMA and acetaldehyde. The potential energy surface was calculated in enzyme environment with the ωB97XD functional and the split-valence double-zeta basis set Def2-SVP by including the MM partial atomic charges into the QM subsystem and performing geometry optimizations for a series of fixed distances between carbinolamine C_1 and N atoms along the reaction coordinate (the distance between C_1 and N). The transition state TS_4 was found at a C_1-N distance of 2.901 Å and has an activation energy of 24.7 kcal/mol making it the rate limiting step of the reaction. The top-right graph shows that the proton from the hydroxyl group of the carbinolamine is transferred to Glu_{491} before reaching the transition state.

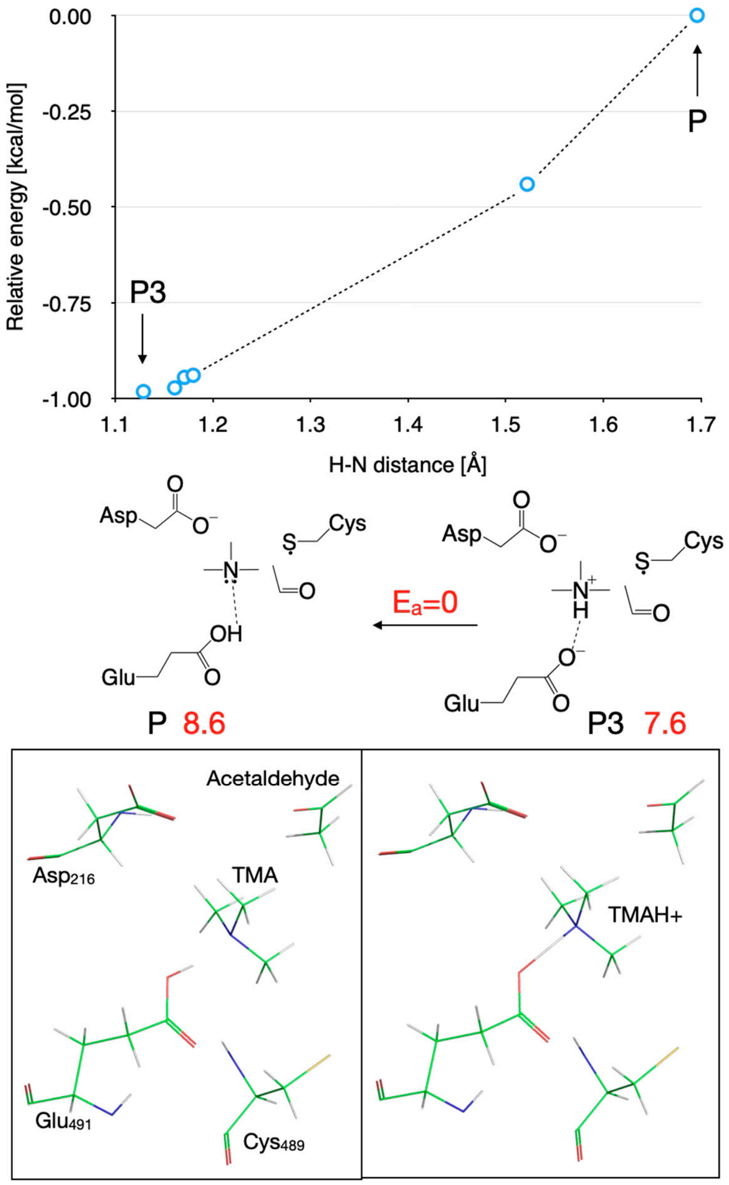

Supplementary Figure S9. Search for the transition state for the proton transfer from Glu_{491} to TMA (TS_5). The potential energy surface was calculated in enzyme environment with the ωB97XD functional and the split-valence double-zeta basis set Def2-SVP without including the MM partial atomic charges into the QM subsystem and performing geometry optimizations for a series of fixed distances between the hydroxylic proton and the N atom of TMA along the reaction coordinate (the distance between H and N). The configuration in which the proton is attached to Glu_{491} (P, bottom-left panel) differs from intermediate 4 (I_4) by the positions of TMA and acetaldehyde and the fact that the hydroxylic proton makes a H-bond with the TMA nitrogen instead with the carbonylic oxygen of acetaldehyde. The PES (relative energy with respect to R) decreases smoothly from 8.6 kcal/mol (P) to 7.6 kcal/mol of the configuration in which the proton is bonded to N of TMA (P3, bottom-right panel) without passing through a transition state, making the activation energy for this reaction step zero.

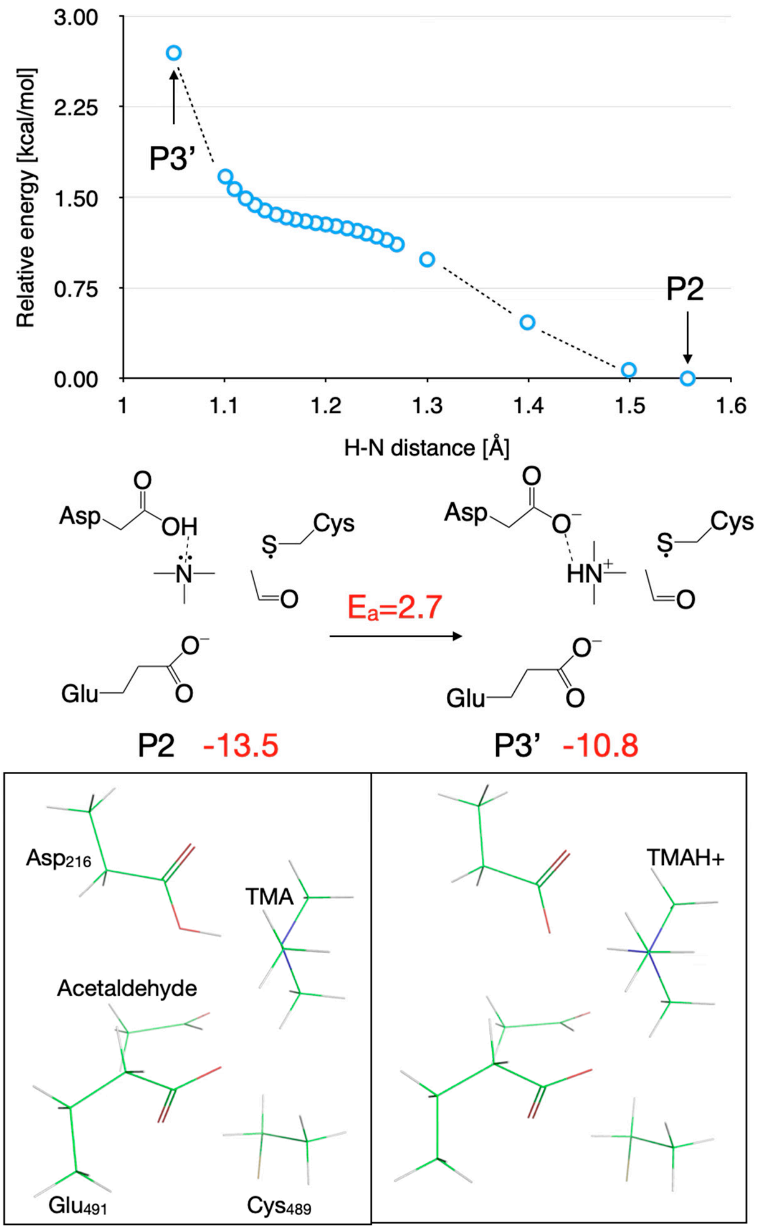

Supplementary Figure S10. Search for the transition state for proton transfer from Asp_{216} to TMA (TS_6). The potential energy surface was calculated in vacuum with the ωB97XD functional and the split-valance double-zeta basis set Def2-SVP and performing geometry optimizations for a series of fixed distances between the hydroxylic proton and the N atom of TMA along the reaction coordinate (the distance between H and N). The configuration in which the proton is attached to Asp_{216} (P2, bottom-left panel) differs from intermediate 4 (I_4) by the positions of TMA and acetaldehyde and the fact that the hydroxylic proton makes a H-bond with the TMA nitrogen instead with the carbonylic oxygen of acetaldehyde. The PES (relative energy with respect to R) increases smoothly from -13.5 kcal/mol (P2) to -10.8 kcal/mol of the configuration in which the proton is bonded to N of TMA (P3', bottom-right panel) without passing through a transition state, making the activation energy for this reaction step equal with its enthalpy (2.7 kcal/mol).

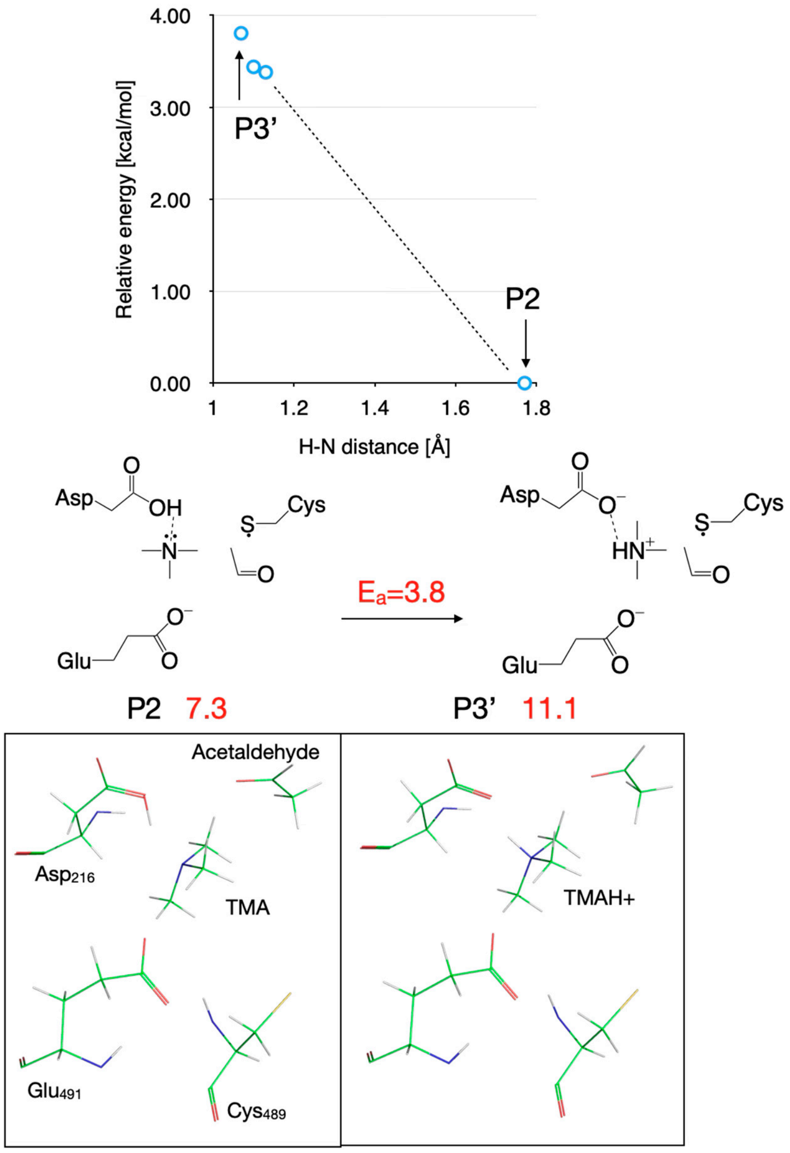

Supplementary Figure S11. Search for the transition state for proton transfer from Aspu$_{216}$ to TMA (TS$_6$). The potential energy surface was calculated in enzyme environment with the ωB97XD functional and the split-valence double-zeta basis set Def2-SVP without including the MM partial atomic charges into the QM subsystem and performing geometry optimizations for a series of fixed distances between the hydroxylic proton and the N atom of TMA along the reaction coordinate (the distance between H and N). The configuration in which the proton is attached to Asp$_{216}$ (P2, bottom-left panel) differs from intermediate 4 (I$_4$) by the positions of TMA and acetaldehyde and the fact that the hydroxylic proton makes a H-bond with the TMA nitrogen instead with the carbonylic oxygen of acetaldehyde. The PES (relative energy with respect to R) increases smoothly from 7.3 kcal/mol (P2) to 11.1 kcal/mol of the configuration in which the proton is bonded to N of TMA (P3', bottom-right panel) without passing through a transition state, making the activation energy for this reaction step equal with its enthalpy (3.8 kcal/mol).

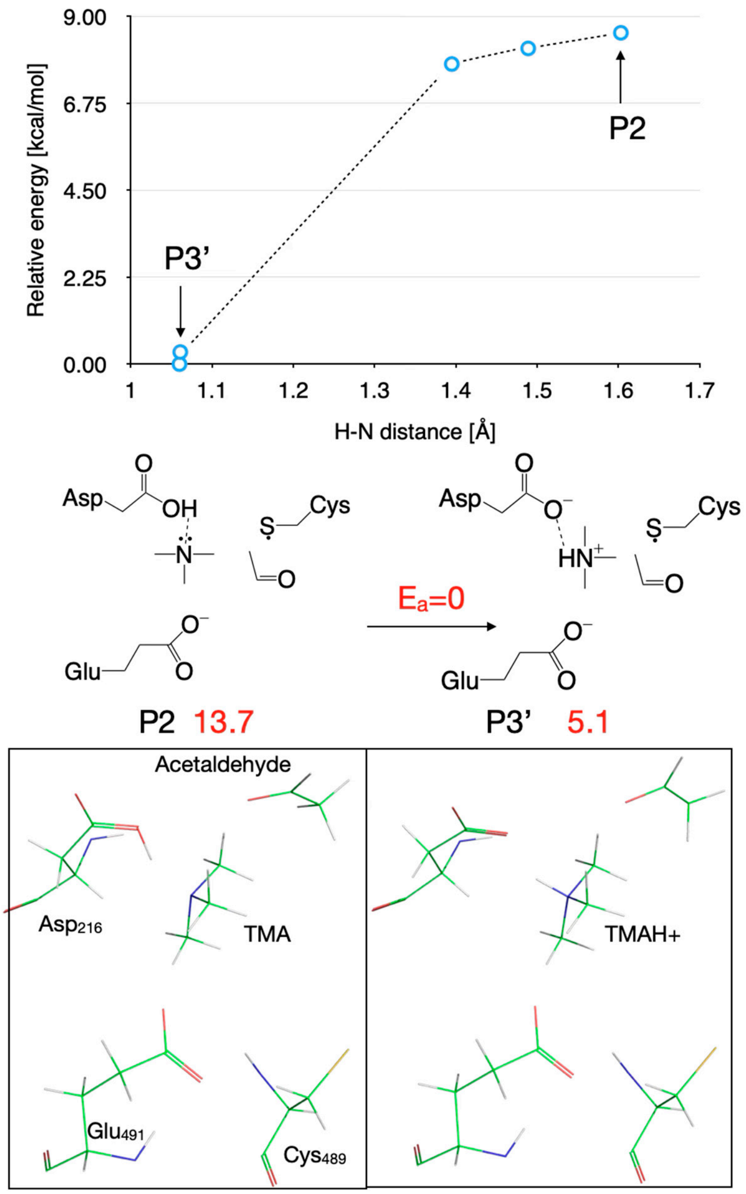

Supplementary Figure S12. Search for the transition state for proton transfer from Aspu$_{216}$ to TMA (TS_6). The potential energy surface was calculated in enzyme environment with the ωB97XD functional and the split-valence double-zeta basis set Def2SVP and by including the MM partial atomic charges into the QM subsystem and performing geometry optimizations for a series of fixed distances between the hydroxylic proton and the N atom of TMA along the reaction coordinate (the distance between H and N). The configuration in which the proton is attached to Asp$_{216}$ (P2, bottom-left panel) differs from intermediate 4 (I_4) by the positions of TMA and acetaldehyde and the fact that the hydroxylic proton makes a H-bond with the TMA nitrogen instead with the carbonylic oxygen of acetaldehyde. The PES (relative energy with respect to R) decreases smoothly from 13.7 kcal/mol (P2) to 5.1 kcal/mol of the configuration in which the proton is bonded to N of TMA (P3’, bottom-right panel) without passing through a transition state, making the activation energy for this reaction step equal to zero.

Supplemental Tables

Supplemental Table S1. Relevant bond distances within intermediate 2 and transition state 3 that discriminate between these structures when calculated in vacuum with various DFT functionals and the split-valence double-zeta basis set Def2-SVP.

Atoms pair	Distance [Å]									
	I_2					TS_3				
	B3LYP	B3LYP-D3	M06-2X	M06-2X-D3[4]	ωB97XD	B3LYP	B3LYP-D3	M06-2X	M06-2X-D3	ωB97XD
Chol-HO…OH	1.536	1.519	1.510	1.049	1.527	1.591	1.566	1.055	1.036	1.605
Chol-HO…OE2-Glu	1.031	1.032	1.024	1.434	1.026	1.018	1.021	1.424	1.476	1.008
Chol-N…C1	3.154	3.032	2.377	1.572	2.958	3.112	2.797	1.584	1.575	2.485
Chol-N…C2	2.460	2.261	2.718	2.509	2.116	2.940	2.831	2.516	2.514	2.724
Chol-C1…H11	-	-	-	-	-	1.421	1.405	1.501	1.606	1.462
Chol-H11…SG-Cys	-	-	-	-	-	1.603	1.605	1.449	1.437	1.516

The structures used in this table for measuring interatomic distances (I_2, TS_3) were obtained through QM calculations in vacuum performed with three different DFT functionals and their variants that include corrections for dispersion interaction. The basis set used was the split-valence double-zeta basis set Def2-SVP (See Methods).

Supplemental Table S2. Electronic energies for reactant, product, reaction intermediates and transition states along the reaction pathway calculated in vacuum and with the QM/MM method using the DFT functional ωB97XD and the split-valence double-zeta basis set Def2-SVP.

Chemical species	Vacuum		QM/MM			
			Without MM charges		With MM charges	
	Energy[a]	$S^{2,b}$	Energy	S^2	Energy	S^2
Reactant	-1379.6701291	0.753	-1380.10348595	0.753	-1920.78894457	0.753
TS_1	-1379.6657298	0.758	-1380.09358693	0.758	-1920.77360291	0.758
I_1	-1379.672712	0.754	-1380.09789546	0.754	-1920.78473115	0.754
TS_2	-1379.6597537	0.763	-1380.09423197	0.757	-1920.76964777	0.755
I_2	-1379.6661777	0.754	-1380.09520944	0.759	-1920.77003717	0.755
TS_2’	-	-	-	-	-1920.76360094	0.760
I_2’	-	-	-	-	-1920.77569013	0.754
TS_3	-1379.6491593	0.768	-1380.07761735	0.763	-1920.75994284	0.759
I_3	-1379.6870706	0.753	-1380.11236099	0.753	-1920.79632770	0.753
TS_4	-1379.6639696	0.753	-1380.09632493	0.753	-1920.75702636	0.753
I_4	-1379.6667167	0.753	-1380.09696341	0.753	-1920.75773901	0.753
P	-1379.6905079	0.753	-1380.08984294	0.753	-1920.77009832	0.753
TS_5	-1379.6870713	0.753	-1380.08984294	0.753	-1920.77009832	0.753
P3	-1379.6837869	0.753	-1380.09140876	0.753	-1920.78305139	0.753
P2	-1380.1657651	0.753	-1380.09179357	0.753	-1920.76709657	0.753
P3’	-1380.1614711	0.753	-1380.08573270	0.753	-1920.76709657	0.753

[a] Total electronic energy of the QM system in hartree.

[b] S^2=S*(S+1) = 0.75 for a radical structure (spin multiplicity=2S+1=2), S=Spin angular momentum quantum number (S=1/2 for electron). The program calculates S^2 by applying the spin angular momentum operator to the QM wavefunction. Deviation of calculated S^2 from the value 0.75 (if the structure contains a single unpaired electron, i.e. multiplicity=2) is a measure of wavefunction spin contamination.

The relative energies and calculated spin squared eigenvalues (S^2) listed in the table are for reactant (R), products (P, P2, P3, P3’), intermediates (I_1, I_2, I_2’, I_3, I_4) and transition states (TS_1, TS_2, TS_2’, TS_3, TS_4, TS_5) found on the reaction pathway of choline-TMA lyase. The structures were obtained through QM vacuum cluster calculations (ωB97XD /Def2-SVP level of QM theory) and through QM/MM calculations (ωB97XD /Def2-SVP level of QM theory and Amber MM force field) in enzyme environment with or without embedding the MM partial atomic charges in the calculation of the QM subsystem (See Methods).
